# Functional Impact and Evolution of a Novel Human Polymorphic Inversion That Disrupts a Gene and Creates a Fusion Transcript

**DOI:** 10.1371/journal.pgen.1005495

**Published:** 2015-10-01

**Authors:** Marta Puig, David Castellano, Lorena Pantano, Carla Giner-Delgado, David Izquierdo, Magdalena Gayà-Vidal, José Ignacio Lucas-Lledó, Tõnu Esko, Chikashi Terao, Fumihiko Matsuda, Mario Cáceres

**Affiliations:** 1 Institut de Biotecnologia i de Biomedicina, Universitat Autònoma de Barcelona, Bellaterra, Barcelona, Spain; 2 Departament de Genètica i de Microbiologia, Universitat Autònoma de Barcelona, Bellaterra, Barcelona, Spain; 3 Estonian Biobank, Estonian Genome Center, University of Tartu, Tartu, Estonia; 4 Boston Children's Hospital, Harvard Medical School, and Broad Institute of Harvard and MIT, Boston, Massachusetts, United States of America; 5 Center for Genomic Medicine, Kyoto University Graduate School of Medicine, Kyoto, Japan; 6 Institució Catalana de Recerca i Estudis Avançats (ICREA), Barcelona, Spain; University of Washington, United States of America

## Abstract

Despite many years of study into inversions, very little is known about their functional consequences, especially in humans. A common hypothesis is that the selective value of inversions stems in part from their effects on nearby genes, although evidence of this in natural populations is almost nonexistent. Here we present a global analysis of a new 415-kb polymorphic inversion that is among the longest ones found in humans and is the first with clear position effects. This inversion is located in chromosome 19 and has been generated by non-homologous end joining between blocks of transposable elements with low identity. PCR genotyping in 541 individuals from eight different human populations allowed the detection of tag SNPs and inversion genotyping in multiple populations worldwide, showing that the inverted allele is mainly found in East Asia with an average frequency of 4.7%. Interestingly, one of the breakpoints disrupts the transcription factor gene *ZNF257*, causing a significant reduction in the total expression level of this gene in lymphoblastoid cell lines. RNA-Seq analysis of the effects of this expression change in standard homozygotes and inversion heterozygotes revealed distinct expression patterns that were validated by quantitative RT-PCR. Moreover, we have found a new fusion transcript that is generated exclusively from inverted chromosomes around one of the breakpoints. Finally, by the analysis of the associated nucleotide variation, we have estimated that the inversion was generated ~40,000–50,000 years ago and, while a neutral evolution cannot be ruled out, its current frequencies are more consistent with those expected for a deleterious variant, although no significant association with phenotypic traits has been found so far.

## Author Summary

Since the discovery of chromosomal inversions almost 100 years ago, how they are maintained in natural populations has been a highly debated issue. One of the hypotheses is that inversion breakpoints could affect genes and modify gene expression levels, although evidence of this came only from laboratory mutants. In humans, a few inversions have been shown to associate with expression differences, but in all cases the molecular causes have remained elusive. Here, we have carried out a complete characterization of a new human polymorphic inversion and determined that it is specific to East Asian populations. In addition, we demonstrate that it disrupts the *ZNF257* gene and, through the translocation of the first exon and regulatory sequences, creates a previously nonexistent fusion transcript, which together are associated to expression changes in several other genes. Finally, we investigate the potential evolutionary and phenotypic consequences of the inversion, and suggest that it is probably deleterious. This is therefore the first example of a natural polymorphic inversion that has position effects and creates a new chimeric gene, contributing to answer an old question in evolutionary biology.

## Introduction

Polymorphic inversions have been known for a long time to segregate in the genomes of multiple species, ranging from insects to plants and animals, and constitute one of the most studied paradigms in evolutionary biology [1–3]. Given that they just change the orientation of a chromosomal segment and often do not result in gain or loss of DNA, inversions could easily be considered neutral variants, but far from this, their adaptive value and phenotypic effects are becoming increasingly clear. For example, in several species of *Drosophila* inversions affect characters like body size or developmental time [2], and some of them exhibit latitudinal or altitudinal clines that strongly suggest adaptation to different environmental conditions [1,4]. Inversions have also been attributed roles in flowering time and reproductive isolation between two ecotypes of monkeyflower [5], wing-pattern morphs in mimetic butterflies [6], stickleback fish freshwater adaptation [7], or in the social behavior and plumage color in sparrows [8], and even a human 900-kb inversion in chromosome 17 is thought to affect fertility and be positively selected in European populations [9].

However, the molecular mechanisms by which inversions are able to affect phenotype are not yet clear. Two main mechanisms have been proposed [10]. One is based on the effective suppression of recombination within the inverted sequence between standard and inverted chromosomes. This suppression can occur both through the reduced pairing and crossing over due to the formation of inversion loops in heterozygotes, and by selection against the unbalanced gametes resulting from unique crossovers within the inverted segment. In this case, the advantage of an inversion could be caused by the capture of a favorable combination of alleles maintained together in strong linkage disequilibrium (LD) due to the reduction of recombination [2,11,12]. Another option is that the phenotypic consequences of inversions depend on the mutational effect of their breakpoints on adjacent genes. Breakpoints can disrupt genes as happens in several disease-causing mutations in humans [13–15] or modify gene expression by separating coding regions from their regulatory elements or by providing new regulatory regions [16,17]. One striking example is the *Rose-comb* mutation in chickens, in which ectopic expression of a gene relocated by a 7.4-Mb inversion causes altered comb morphology and a gene disrupted by the same inversion has been associated to poor sperm motility [18]. Also, in humans, certain inversions mediated by repeats of complex structure seem to predispose to microdeletions that cause genetic disorders in the offspring [10,19,20], although in these cases, the disease-causing mutation is the deletion rather than the inversion. Nevertheless, very few cases of position effects have been identified for polymorphic inversions in natural populations [18,21].

Inversions represent a special challenge in complex genomes such as that of humans, not only due to their balanced nature, but also because their breakpoints are usually located within highly identical inverted repeated sequences that can reach large sizes (>100 kb). For these reasons, inversions have often been set aside in favor of other structural variants that are easier to detect, like copy number variants (CNVs). In recent years, the use of high-throughput techniques like paired-end mapping [22–24] has allowed the detection of multiple candidate regions to contain inversions in the human genome, but it is necessary to validate them since many represent different types of errors rather than real polymorphic variants [25]. Besides, large projects that try to characterize all human variation like the 1000 Genomes Project (1000GP) [26] might miss many inversions, either because short next-generation sequencing reads and paired-ends are not able to cross long repeats causing inversions [27], or due to the low coverage and difficulty of mapping reads containing simple breakpoint sequences.

So far, only a few polymorphic inversions not directly related to pathological processes have been studied in detail in humans [9,28–30]. Some of them have been associated to different expression levels of genes contained within the inverted sequence, such as the polymorphic inversion in chromosome 17q21 that is associated to a decreased *MAPT* expression, among other expression changes [31], or the 4.5-Mb inversion at 8p23 [29] and the 0.45-Mb inversion at chromosome 16p11 [32], where computationally-predicted inversion alleles correlate with expression levels of several genes. However, we do not know if these expression changes are consequence of position effects caused by the inversion breakpoints or of specific nucleotide changes captured by the inversion. These inversions have also been associated with certain phenotypes, such as recombination, fertility and several neurodegenerative diseases for the 17q21 inversion [9,33,34], or asthma and obesity for the 16p11 inversion [32], but the mechanisms linking the differentially expressed genes in the inverted region to their phenotypic consequences have not been completely elucidated. Other inversions have breakpoints located in positions where effects on adjacent genes would be expected. This is the case of a recently-described 16.5-kb polymorphic inversion that disrupts chymotrypsinogen precursor genes *CTRB1* and *CTRB2* by exchanging their respective first exons [30], although as both genes belong to the same family, complete copies are reconstructed in the inverted arrangement.

In this work, we describe a new polymorphic inversion found at a 4.7% frequency in East Asians, which represents one of the few inversions genotyped in several worldwide populations that show a limited geographical distribution. In addition, the inversion disrupts and inactivates a transcription factor gene and generates a new transcript in inverted chromosomes, thus providing mechanisms by which it may be directly affecting phenotype. Finally, we show that these changes are able to generate distinct genome-wide expression patterns and investigate the inversion’s functional and evolutionary impact.

## Results

### Identification of inversion breakpoints

**Fig 1:**
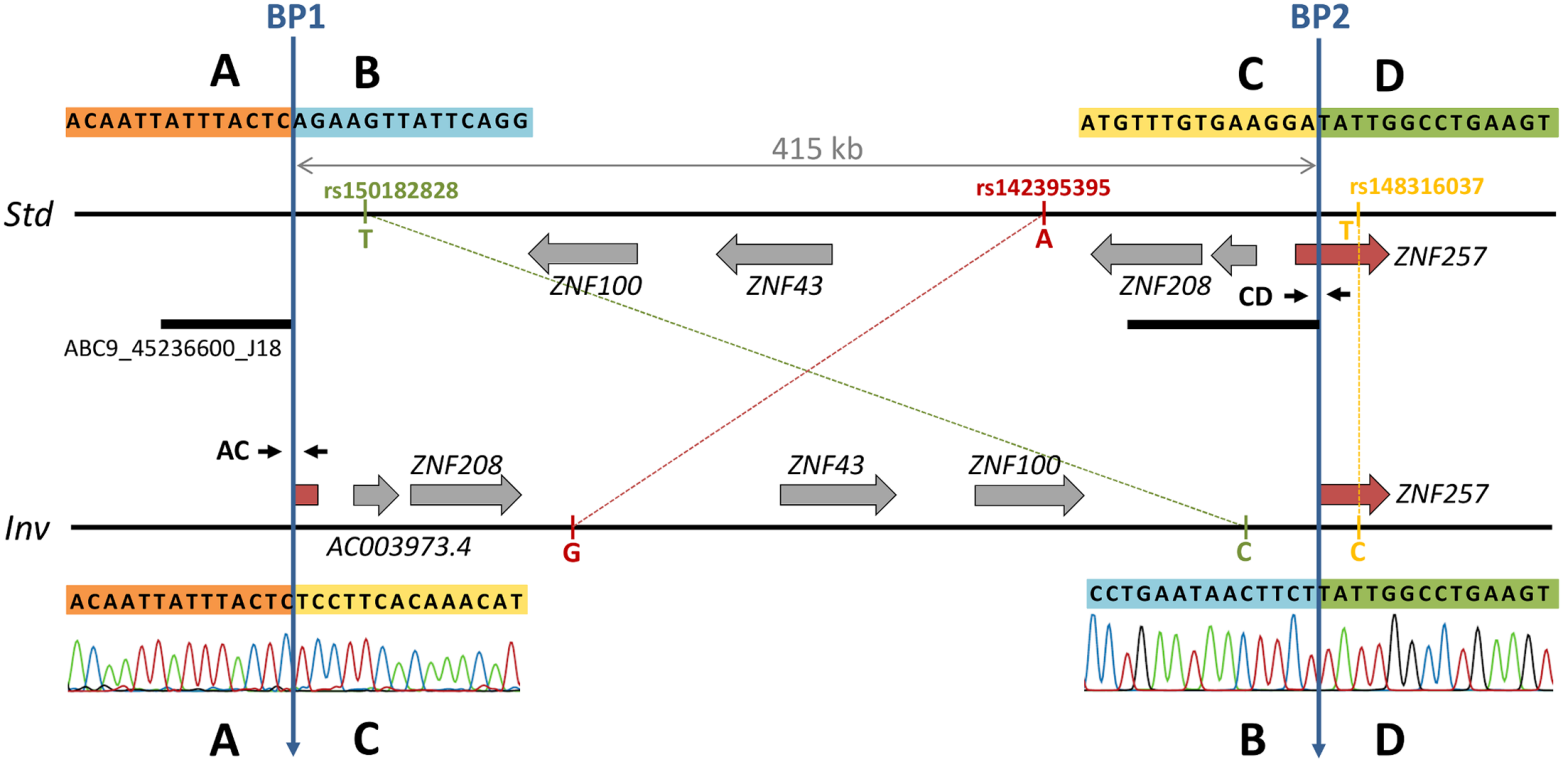
Inversion features and genotyping strategies. Horizontal lines represent the standard (*Std*, top) and inverted (*Inv*, bottom) arrangements. Genes are depicted as grey arrows indicating direction of transcription with the disrupted gene shown in red. Blue vertical arrows mark the two inversion breakpoints (BP1 and BP2). The black bar below the *Std* chromosome indicates the sequence included in the analyzed fosmid containing BP1 in the inverted orientation. Primers used for PCR genotyping are shown in the corresponding breakpoint of each arrangement as small black arrows. The three tag SNP alleles are shown next to the corresponding chromosome. Sanger chromatograms show the sequence of inversion breakpoints in the *Inv* chromosome.

Using available fosmid paired-end mapping data in humans [22], HsInv0379 inversion was predicted in chromosomal band 19p12 in a region close to the centromere [25]. This inversion was supported by five discordant fosmid clones belonging to a Japanese individual, NA18956, who also has concordant clones in the same genomic region. The analysis of available partial sequences from fosmid ABC9-45236600J18 insert obtained by 454 shotgun sequencing confirmed the presence of an inversion breakpoint, since these sequences map in two different regions separated by more than 400 kb on the GRCh37 (HG19) reference genome (Fig 1). Therefore, a fragment of 7 kb including the inversion breakpoint 1 (BP1) was amplified by PCR from the fosmid DNA and analyzed by restriction mapping with several enzymes (S1 Fig), which allowed us to locate the breakpoint within a 1.5-kb segment. Primers flanking this region were then used to amplify and sequence a 1,894-bp band containing the breakpoint directly from individual NA18956 genomic DNA. The alignment of this sequence in the inverted chromosome (*Inv*) with the reference genome (*Std*) revealed BP1 exact position, defining regions A (outside the inversion) and B and C (inside the inversion) (Fig 1). The mapping in the reference genome of sequence C allowed us to locate breakpoint 2 (BP2) and an 850-bp BD PCR product was amplified and sequenced from NA18956 genomic DNA.

The analysis of BP1 and BP2 sequences revealed clean breaks with no insertions or deletions that were located at HG19 chr19 positions 21830133–21830134 (BP1) and 22245260–22245261 (BP2), resulting in a 415-kb inversion (Fig 1). BP1 falls within the first copy (42.9-kb long) of a pair of inverted segmental duplications located 24.5 kb apart, although they show only a 91.6% identity and present multiple indels that make the mapping of BP1 unequivocal. Both inversion breakpoints are located within blocks of transposable elements (TEs) of 14.3 kb in BP1 and 4.9 kb in BP2 formed by a LINE element with several *Alu* sequences inserted inside at different positions (S1 Fig). Even though the composition of both blocks is similar, the nucleotide identity between them is very low and the inversion breakpoints occurred within *L1* elements of different subfamilies in a direct orientation with respect to each other, which suggests that the inversion was not generated by non-allelic homologous recombination (NAHR). No microhomology was detected at the breakpoints either. Therefore, the most plausible generation mechanism for this inversion is non-homologous end joining (NHEJ). These TE blocks also indicate that the *Std* chromosome corresponds to the ancestral arrangement because the *L1* copies are abruptly interrupted at the breakpoint in the derived *Inv* sequence (S1 Fig). In addition, the *Std* arrangement is found in the chimpanzee genome sequence (PanTro4 assembly).

### Inversion frequency and geographical distribution

To find out its distribution and frequency in different human populations, inversion HsInv0379 has been genotyped using three different strategies (Table 1). First, 534 individuals from seven human HapMap populations (CEU and TSI of European origin, YRI and LWK of African origin, and GIH, CHB and JPT of Asian origin; see Table 1 for details) were genotyped by multiplex PCR across the breakpoints (Fig 1 and S1 Fig). Only five individuals carrying the inversion in heterozygosis were found, all in East Asian populations (ASN), two CHB (2.2% *Inv* frequency) and three JPT (3.3% *Inv* frequency), and inversion genotypes fit perfectly Hardy-Weinberg equilibrium (*P* = 0.4636).

**Table 1:** HsInv0379 genotyping and frequency in different populations. Number of individuals genotyped by PCR across inversion breakpoints, tag SNPs in high linkage disequilibrium with the inversion, and sequence reads spanning inversion breakpoints (BreakSeq). The number of unrelated individuals genotyped is also given together with genotype counts and allelic frequencies for each analyzed population.

Population code	Population description (Sample panel)	PCR	Tag SNPs	BreakSeq	Genotyped unrelated	*Std*/*Std*	*Std*/*Inv*	*Inv*/*Inv*	*Inv* freq
**East Asian (ASN)**									
CHB	Han Chinese in Beijing, China (HapMapPT02)	48	105	62	110	104	6	0	**0.0273**
JPT	Japanese in Tokyo, Japan (HapMapPT02)	47	105	47	104	99	5	0	**0.0240**
CHS	Southern Han Chinese	0	113	31	111	98	13	0	**0.0586**
CDX	Chinese Dai in Xishuangbanna, China	1	99	13	93	79	13	1	**0.0806**
KHV	Kinh in Ho Chi Minh City, Vietnam	0	101	2	99	87	12	0	**0.0606**
Malay	Malays from Singapore	0	96	0	96	89	7	0	**0.0365**
**South Asian (SAN)**									
GIH	Gujarati Indian from Houston, Texas (HapMapV15)	90	106	0	109	109	0	0	0
PJL	Punjabi from Lahore, Pakistan	0	96	0	96	96	0	0	0
BEB	Bengali from Bangladesh	0	86	0	86	85	1	0	**0.0058**
STU	Sri Lankan Tamil from the UK	0	103	0	101	101	0	0	0
ITU	Indian Telugu from the UK	0	103	0	102	102	0	0	0
**European (EUR)**									
CEU	Utah Residents (CEPH) with Northern and Western European ancestry (HapMapPT01)	90	103	62	115	115	0	0	0
TSI	Toscani in Italia (HapMapV14)	88	110	87	109	109	0	0	0
FIN	Finnish in Finland	0	103	22	103	103	0	0	0
GBR	British in England and Scotland	0	99	32	98	98	0	0	0
IBS	Iberian population in Spain	0	107	1	107	107	0	0	0
**African (AFR)**									
YRI	Yoruba in Ibadan, Nigeria (HapMapPT03)	89	111	58	120	120	0	0	0
LWK	Luhya in Webuye, Kenya (HapMapV12)	88	115	9	113	113	0	0	0
ASW	Americans of African Ancestry in Southwest USA	0	68	23	59	59	0	0	0
GWD	Gambian in Western Divisions in The Gambia	0	113	0	113	113	0	0	0
MSL	Mende in Sierra Leone	0	85	0	85	85	0	0	0
ESN	Esan in Nigeria	0	99	0	99	99	0	0	0
ACB	African Caribbeans in Barbados	0	96	2	86	86	0	0	0
**American (AMR)**									
MXL	Mexican Ancestry from Los Angeles, USA	0	71	25	68	67	1	0	**0.0074**
PUR	Puerto Ricans from Puerto Rico	0	105	6	104	104	0	0	0
CLM	Colombians from Medellin, Colombia	0	96	0	96	96	0	0	0
PEL	Peruvians from Lima, Peru	0	86	0	85	85	0	0	0
**TOTAL**	**All populations**	**541**	**2680**	**482**	**2667**	**2608**	**58**	**1**	**0.0112**
**TOTAL**	**East Asian populations**	**96**	**619**	**155**	**613**	**556**	**56**	**1**	**0.0473**

Second, taking advantage of the fact that many individuals genotyped by PCR had been sequenced in the 1000GP Phase 1 [26], we searched for SNPs in LD with the inversion. The comparison of the SNP genotypes in the inverted segment plus 20 kb of flanking sequence revealed three tag SNPs (rs150182828, rs142395395 and rs148316037) with alleles that segregate in complete LD with the inversion (*r* ^2^ = 1). For these three SNPs, which span 400.8 kb, all *Std* chromosomes carry the ancestral reference alleles (Fig 1) while the five heterozygote individuals are the only ones with the alternative alleles, also in heterozygosis. Two tag SNPs are located within the inverted sequence in intergenic regions and the third one right outside, in an intron of gene *ZNF257* (Fig 1).

The analysis of the recently released SNP calls for 2,535 genomes included in 1000GP Phase 3 (ftp://ftp.1000genomes.ebi.ac.uk/vol1/ftp/release/20130502/) together with the already published 1,092 genomes in Phase 1 [26], and 96 Malay genomes from the Singapore Sequencing Malay Project (SSMP) [35] allowed us to identify 56 additional candidate individuals to carry the inversion based on the tag SNPs (Table 1 and S1 Table). All these individuals carry the alternative alleles for the three SNPs except five individuals, which have only two of the alleles, and HG02152 (CDX), that is homozygous for two alternative SNP alleles. The predicted inversion genotypes were confirmed by PCR in six of the new heterozygotes and the *Inv* homozygote. Interestingly, almost all the potential inversion carriers belong to East Asian populations (CHB, JPT, CHS, CDX, KHV and Malays), but we also identified a Bengali (BEB) and a Mexican (MXL) individual that are clearly heterozygous for the three tag SNPs (S1 Table).

**Fig 2:**
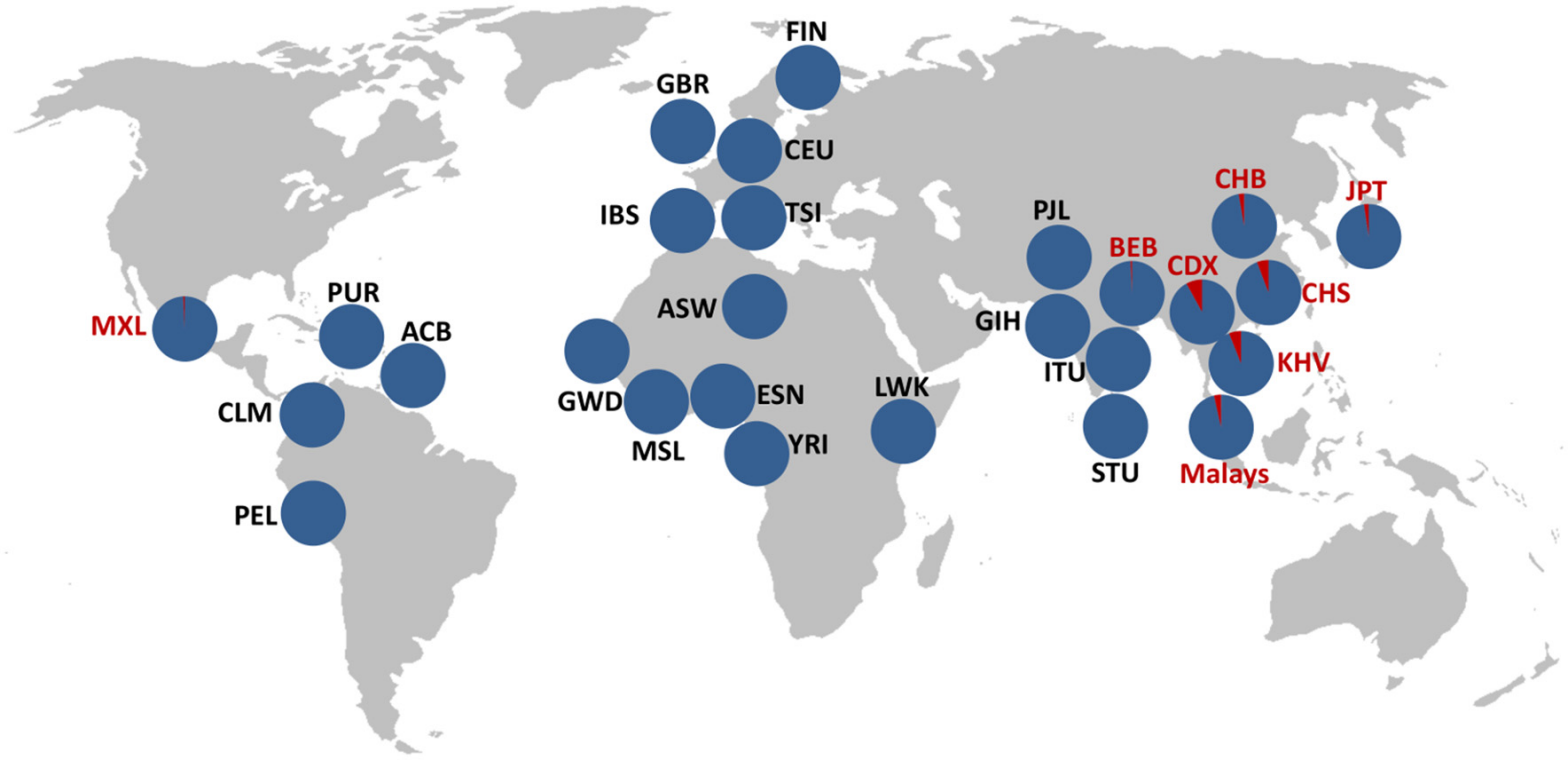
Geographical distribution of HsInv0379 inversion. Inversion frequency in 27 different populations corresponding to 2,667 unrelated individuals analyzed by PCR, tag SNPs and reads spanning inversion breakpoints are shown. Blue corresponds to *Std* and red to *Inv* arrangement. Inversion frequencies and the origin of the different populations are listed in Table 1.

Finally, we performed similarity searches against the reads of 1,892 individuals from 19 populations available at the 1000GP ftp site on summer 2013 (including both mapped and unmapped reads) using 100-bp sequences spanning the two breakpoints in each arrangement as queries (S2 Table). Reads containing at least one of the inversion breakpoints (from either *Std* or *Inv* orientation) were retrieved for 482 individuals, with an average of just 3.6 reads/individual due to the low coverage of many of these genomes. Of those, *Inv* chromosomes were detected in 19 of 20 candidate *Std*/*Inv* heterozygotes according to the tag SNPs (the remaining individual having one *Std* read), whereas only reads corresponding to the *Std* arrangement were recovered from the 462 predicted *Std*/*Std* individuals (S1 Table). *Std* alleles were also detected in nine of the inversion carriers, validating their heterozygote status. In total, we have independent PCR and/or breakpoint-sequence confirmation of the *Inv* allele for 25 of the 61 individuals (41%) predicted by the tag SNPs to carry the inversion (59 unrelated; S1 Table). Therefore, we can confidently use these SNPs as proxies to detect *Inv* chromosomes. Overall, inversion genotypes were determined for 2,771 individuals (2,667 excluding related individuals; Table 1 and S1 Table), of which 197 individuals from different populations have been genotyped by the three methods and results are in complete agreement. These genotypes reveal an inversion frequency of 1.12% worldwide and of 4.73% in East Asia, which ranges from ~2.5% in the Northern populations to ~7% in the Southern populations (being Malays an exception with a frequency of 3.65%) (Table 1 and Fig 2).

### Functional consequences of the inversion

**Fig 3:**
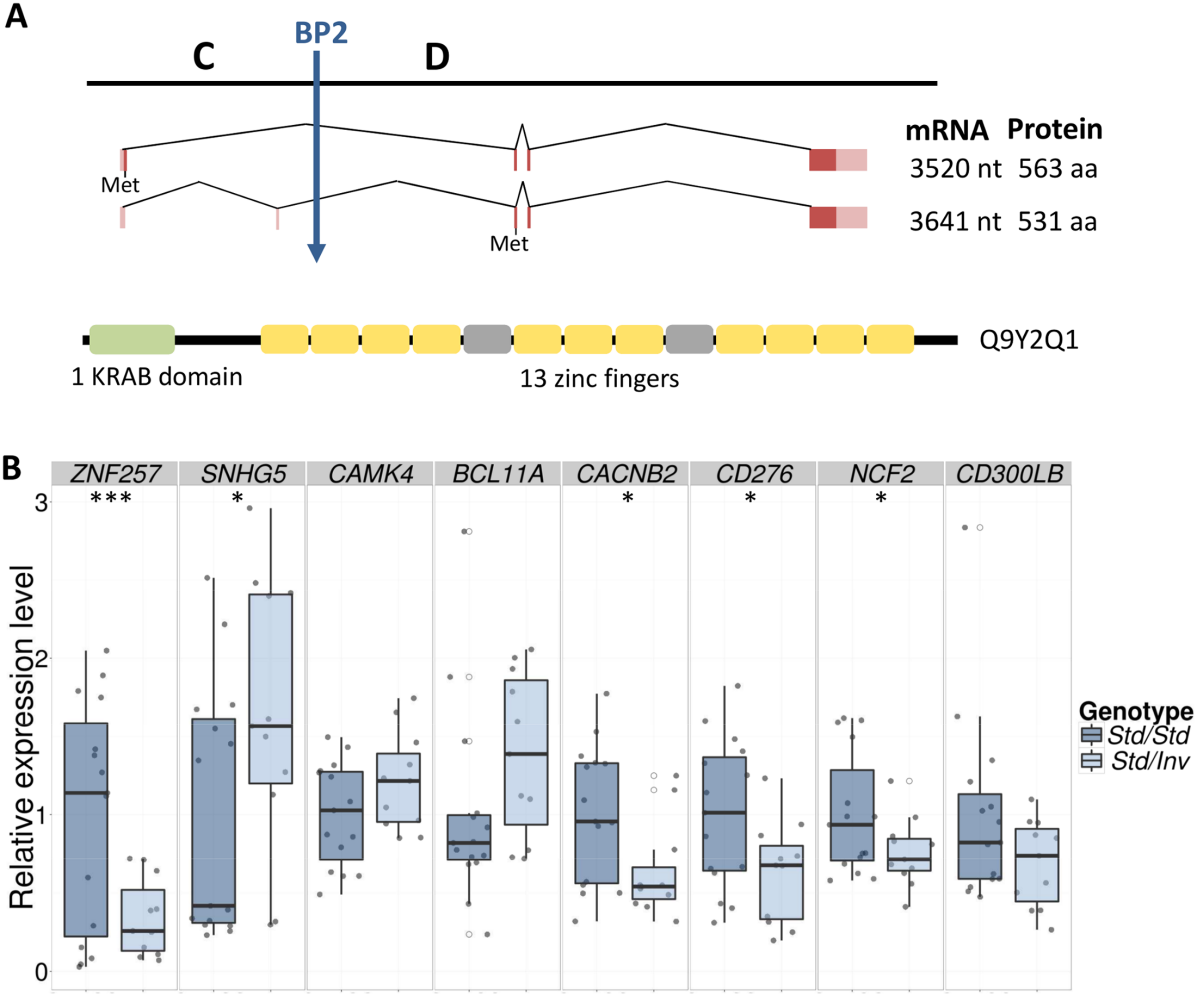
*ZNF257* gene structure and expression changes in inversion carriers. **A.** The two most reliable transcripts of gene *ZNF257* are represented as dark and light-colored boxes corresponding to coding and non-coding exons, with their sizes and encoded proteins shown to the right. The longer transcript includes an extra 121-bp alternative non-coding exon in its 5’ UTR and in theory encodes a 32-aa shorter protein because of the shift in the methionine (Met) used as translation initiation from the three last nucleotides of the first exon to the third exon, common to both isoforms. The position of BP2 is indicated by a vertical arrow. A diagram of the longest protein domains (UniProt Q9Y2Q1) is represented by boxes below: green for the *Krüppel*-associated box (KRAB) domain, yellow for C2H2-type zinc fingers, and grey for degenerate zinc finger structures. **B.** Box plots of qPCR expression levels for *ZNF257* and the seven genes with significant or marginally significant differences between LCLs of 15 *Std*/*Std* (dark blue) and 11 *Std*/*Inv* (light blue) individuals. For each gene, expression values have been normalized by the average of *Std*/*Std* individuals, with every sample represented by grey points and outliers by open circles. Horizontal black lines within each box indicate median values. *, *P* < 0.05; ***, *P* < 0.001.

The HsInv0379 inverted segment contains three protein-coding genes: *ZNF100*, *ZNF43* and *ZNF208* and at least one long intergenic non-coding RNA (reported in GENCODE v19 gene annotation). A fourth protein-coding gene, *ZNF257*, appears to be disrupted by BP2 (Fig 1). *ZNF257* encodes a transcription factor with a *Krüppel*-associated box (KRAB) repressor domain and 13 zinc fingers. This gene has two main transcripts of 3,520 and 3,641 nucleotides, which are transcribed from the same promoter but differ in the presence of an extra non-coding second exon (Fig 3). The inversion removes the first two exons as well as the promoter of this gene, which are relocated 415 kb away in the *Inv* chromosomes. Thus, total *ZNF257* expression (both isoforms included) was quantified by quantitative PCR (qPCR) in lymphoblastoid cell lines (LCLs) of 15 *Std*/*Std* and 11 *Std*/*Inv* individuals from the CHB and JPT populations, plus the *Inv/Inv* homozygote (CDX). A 2.9-fold decrease (*P* = 0.0047) was found in the inversion carriers compared to the homozygote *Std* individuals and no expression was detected in the *Inv/Inv* individual (Fig 3 and S2 Fig), indicating that, as expected, this gene is not expressed from the *Inv* chromosomes. However, five *Std*/*Std* individuals showed an extremely low *ZNF257* expression, comparable to the levels in inversion carriers (S2 Fig). These individuals are all from the CHB population and most likely carry some regulatory variant in *cis* or *trans* that causes a lower expression of *ZNF257* not related to the presence of the inversion. In fact, an analysis of Geuvadis RNA-Seq data [36] in LCLs from European and YRI populations (which include only *Std/Std* homozygotes) indicates that *ZNF257* is among the top 10% genes with highest variation in expression among those with similar low expression levels (S3 Fig). In addition, several studies including the recent results from the GTEx project [36,37] suggest the existence of eQTLs for the gene, with 13 SNPs significantly associated to *ZNF257* expression in different tissues, although due to the transformation of the data during the analysis the biological interpretation of the observed effects is not easy. In our data set, each of these SNPs explains 9.8–19.8% of the variation of *ZNF257* expression in *Std*/*Std* homozygotes, which contrasts with the 27.6% explained by the inversion across all the samples assuming an additive model (S3 Table).

Since *ZNF257* is a transcription factor that may be controlling the expression of other genes, we searched for genome-wide expression changes associated to the inversion by analyzing the RNA-Seq profiles of LCLs in eight of the previous *Std*/*Std* and *Std*/*Inv* individuals showing discrepant levels of *ZNF257* expression (see S4 Table for RNA-Seq summary results). We detected 56 differentially expressed protein-coding genes and 27 non-coding RNAs (False Discovery Rate, FDR < 0.1), of which 49 were down-regulated and 34 up-regulated, although 60 of them (72.3%) have an FDR < 0.05. These genes exhibit moderate to high fold changes (1.2–8.7) with the greater variation corresponding to non-coding RNAs (S5 Table), and none of them are located within or close to the inverted sequence. Because only four individuals of each genotype were compared, the results could be confounded by differences between the two groups other than the presence of the inversion. Thus, we repeated the analysis for the 18 possible permutations exchanging two individuals from each genotype class (S4 Fig). The median proportion of genes differentially expressed between the two groups in the permutations was 0.17%, which is ~3 times less than those identified between *Std/Std* and *Std/Inv* individuals using the same criteria (0.47%, at a nominal FDR < 0.1). Besides, none of the genes identified in the permutations were in the differentially expressed list from the inversion genotype comparison. We also performed 400 simulations of read counts with the mean and dispersion of the real data and searched for differentially expressed genes with FDR < 0.1 in each case. Only 5.5% of the simulations had a higher number of differentially expressed genes than the comparison of individuals with different inversion genotypes.

Next, several enrichment analysis tools (see Methods) were used to try to detect functional relationships among the differentially expressed genes, but no statistically significant functional categories have been uncovered based on current knowledge. However, immune response was the most significant biological process gene-ontology category in the DAVID Functional Annotation Tool (*P* = 0.00091), although significance was lost after correcting for multiple testing. Thus, we selected 11 genes involved in the immune system with different fold-change values and FDRs to validate their expression in the whole set of available LCL RNAs (15 *Std/Std*, 11 *Std/Inv* and 1 *Inv/Inv*) using qPCR. All the genes except one showed good validation when considering only the eight individuals used in the RNA-Seq analysis (Table 2). In the large sample set, the expression difference was always in the expected direction (up or down regulated) except for one gene (*NFATC1*), but qPCR validation depended on the gene’s FDR. For FDR < 0.05, significant differences between *Std/Std* and *Std/Inv* were obtained for three genes, while the other three were marginally significant (Table 2 and Fig 3). Of the three genes with FDR = 0.05–0.10, only the expression difference of the *CACNB2* gene (FDR = 0.077) could be validated, while none of the two genes tested with higher FDR showed significant differences. As for *ZNF257*, *w*e also see a significant or marginally-significant correlation between the expression levels and inversion genotype (*Std/Std* or *Std/Inv*) for five genes (Table 2), in which inversion genotype explains between 10% and 19% of the variation in gene expression. Therefore, there is a clear association between inversion genotypes and gene expression of several genes in *trans*. In the case of the *Inv/Inv* sample, it behaves as expected from the expression change observed in heterozygotes in 7 of the 11 genes tested, although since expression levels for any given gene can be affected by many variables, it is difficult to draw reliable conclusions with a single individual.

**Table 2:** Analysis of RNA-Seq gene-expression changes between *Std/Std* and *Std/Inv* individuals by qPCR. For each gene, fold change and differential expression p-value (*P*) between inversion genotypes are given both for only the eight samples also analyzed by RNA-Seq and for the total 26 samples, with the RNA-Seq results shown for comparison (a line separating genes with FDR < 0.05 from those with lower significance). *R* ^*2*^ represents the percentage of gene-expression variation explained by the inversion genotype according to an additive model, and *R* the Pearson's correlation between the expression levels of *ZNF257* and each analyzed gene.

Gene			qPCR					
	RNA-Seq		RNA-Seq samples		All samples		*R* ^*2*^	*R*
	Fold change	FDR	Fold change	*P* ^1^	Fold change	*P* ^1^	Inversion	*ZNF257*
*SNHG5*	4.02	8.34 x 10^−16^	5.47	0.0012**	1.63	0.0343*	0.1316^ms^	-0.3866^ms^
*NCF2*	-1.86	7.61 x 10^−7^	-1.66	0.0561^ms^	-1.32	0.0388*	0.1241^ms^	0.2691
*CAMK4*	1.84	1.66 x 10^−6^	2.14	0.0029**	1.21	0.0593^ms^	0.09845^ms^	-0.1532
*BCL11A*	1.81	2.41 x 10^−5^	2.10	0.0158*	1.38	0.0585^ms^	0.0993	-0.3805^ms^
*CD276*	-1.74	4.62 x 10^−5^	-4.88	0.0014**	-1.58	0.0183*	0.1697*	0.2438
*CD300LB*	-1.47	0.0111	-3.55	0.0334*	-1.45	0.0642^ms^	0.0936	0.2188
*GCNT3*	-1.38	0.0707	-2.83	0.0335*	-1.21	0.2134	0.0265	0.2490
*CACNB2*	-1.47	0.0777	-1.63	0.0429*	-1.57	0.0134*	0.1884*	-0.0722
*PIK3R5*	1.46	0.0838	1.57	0.0509^ms^	1.10	0.2413	0.0208	-0.3045
*NFATC1*	-1.38	0.2167	-1.62	0.0138*	1.04	0.4087	0.0023	-0.1655
*IL4R*	1.28	0.3096	1.25	0.1326	1.11	0.1433	0.0472	-0.1920

^1^ Probabilities of the qPCR validation of the RNA-Seq expression differences are calculated using a one-tailed Student *t*-test

^ms^, *P* < 0.1

*, *P* < 0.05

**, *P* < 0.01

***, P < 0.001.

Since we found five *Std/Std* individuals with low expression levels of *ZNF257* mRNA, we have an opportunity to test the relationship between this gene and the detected expression changes. A marginally significant correlation between the *ZNF257* expression values and that of the other genes tested by qPCR in the 26 *Std/Std* or *Std/Inv* individuals was found for two genes, *SNHG5* and *BCL11A* (Table 2), which indicates that a low *ZNF257* expression level is associated to an increase in the two genes. Accordingly, for these genes the significance of the expression change between *Std/Std* and *Std/Inv* is higher when the five *Std/Std* with low *ZNF257* expression are removed from the homozygous *Std* group (*P* = 0.0140 and *P* = 0.0096, respectively). Conversely, this does not happen for other genes like *CACNB2*, which shows no clear association with *ZNF257*, suggesting that these expression changes depend more on inversion genotype than on *ZNF257* level.

**Fig 4:**
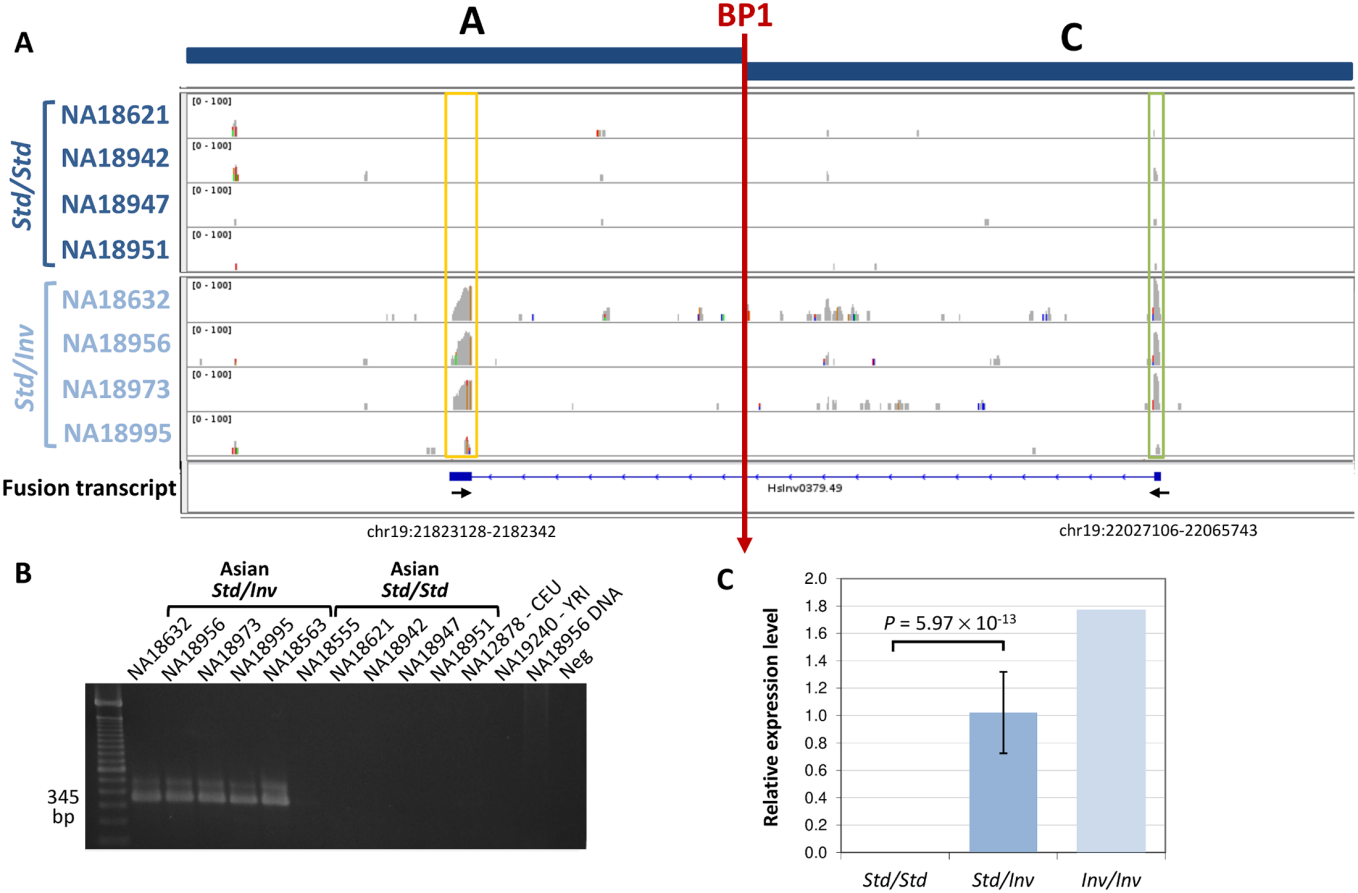
Analysis of the new fusion transcript in HsInv0379 breakpoint. **A.** RNA-Seq reads mapped to an AC construct corresponding to BP1 (vertical red arrow) in an inverted chromosome reveal a fusion transcript present only in the four *Std*/*Inv* individuals (bottom) but not in the four *Std*/*Std* (top). Boxes highlight the new exon (yellow) and *ZNF257* first exon (green). The structure of the fusion transcript reconstructed by Cufflinks [38] is shown below the RNA-Seq profiles. Small arrows indicate the approximate position of the primers used to validate the transcript. The coordinates of the new exon in the HG19 reference genome are also indicated. **B.** Analysis of fusion transcript expression by RT-PCR in several individuals. **C.** Quantification of the fusion transcript levels by qPCR in 15 *Std*/*Std*, 11 *Std*/*Inv*, and 1 *Inv/Inv* individuals.

Another important effect of the inversion is that it brings the *ZNF257* promoter and first exons to a completely different part of the genome, where they might induce the transcription of new chimeric transcripts. Therefore, we specifically searched for transcripts generated by the *ZNF257* promoter around BP1 in *Inv* chromosomes. By mapping RNA-Seq reads to a construct with the inverted sequence, we detected a spliced fusion transcript formed by the first exon of gene *ZNF257* and a completely new 296-bp exon made up of fragments of LINE and *Alu* elements (Fig 4). We confirmed the existence of this RNA by amplifying most of it by RT-PCR and by sequencing the exon-exon junction in individual NA18956. Indeed, both by RT-PCR and qPCR the fusion transcript was found in the 11 inversion carriers analyzed, with 1.7-fold higher expression levels in the *Inv/Inv* individual, while it is not expressed in any of the *Std*/*Std* individuals tested (including three non-East Asian individuals) (Fig 4 and S2 Fig). The complete new transcript is short, with only 468 bases, and its longest ORF contains only 75 aa. It does not show homology to any other known transcript either. However, according to the RNA-Seq reads mapping to the *ZNF257* exon 1 (common to the two genes) in inversion heterozygotes that express both transcripts and in *Std* homozygotes (Fig 4), the level of expression of the fusion transcript is at least 7.4 times higher than that of *ZNF257*.

Because the inversion tag SNPs are not included in SNP genotyping arrays, the potential functional effects of the inversion have been missed by typical genome-wide association studies. Therefore, based on the lymphoblast transcriptomic results, we made an attempt to associate the inversion allele with general blood-related phenotypic traits by genotyping the inversion tag SNP rs148316037 in 3,787 Japanese individuals of the Nagahama cohort [39–41]. The inversion showed a frequency of 1.95% in this population with a single homozygote and 146 heterozygotes, which fits perfectly the Hardy-Weinberg equilibrium (*P* = 0.71). A regression analysis was performed to assess the effects of the inversion on immunological, hematological and metabolic measures from blood samples available for this cohort (S6 Table). However, no significant associations have been detected between HsInv0379 inversion alleles and the phenotypical traits analyzed, including disease indicators for diabetes and cardiovascular or liver disease, as well as alterations in blood cell counts or presence of certain antibodies (S6 Table). Only red blood cell count shows a decrease in inversion carriers (*P* = 0.036), but this association is lost after multiple testing correction.

### Origin and evolutionary history of the inversion

The presence of tag SNPs and identical breakpoints in the reads of all *Inv* chromosomes indicate that the inversion was generated only once from the ancestral *Std* orientation. Thus, to investigate the evolutionary history of the inversion, we first estimated the age of the derived *Inv* allele. We used in this analysis the unphased Malay population SNP data set from the SSMP [35] because it provides accurate SNP genotypes for 96 individuals based on >30x coverage sequencing. In 381,827 bp of the inverted region we identified 30 SNPs that are polymorphic exclusively in the seven *Std*/*Inv* heterozygotes (out of 2,005 SNPs without missing values). Of these, one corresponds to the tag SNP rs142395395 and four SNPs are found only in three heterozygotes, so they most likely represent variants specific of the *Inv* chromosomes (the probability that one such variant belongs to *Std* chromosomes is *P* = 0.0001). The remaining 25 variants are singletons, and 22 were attributed to the seven *Std* chromosomes in heterozygotes (on average there are 3.12 singletons/chromosome), leaving 3 singletons in *Inv* chromosomes. Based on this variation, we determined a nucleotide diversity (π) of 8.86 × 10^−4^ for *Std* and 9.73 × 10^−6^ for *Inv* chromosomes. We used the divergence between the human and chimpanzee genomes (d_H-C_ = 0.0134) to establish a local substitution rate for this region. Taking into account this substitution rate and the pairwise divergence between *Std* and *Inv* chromosomes (d_S-I_ = 9.95 × 10^−4^), after subtracting the variation among ancestral *Std* haplotypes [42], we estimated the age of the inversion in 43,450 years (95% confidence interval (CI): 14,934–93,438) or 52,140 years (95% CI: 17,921–112,126), assuming a divergence time between human and chimpanzee of 5 or 6 mya, respectively.

Next, to understand better the effects of the inversion on the nucleotide variation patterns, we phased the SNPs for a 4-Mb region around the inverted segment in the 286 1000GP Phase 1 East Asian individuals (97 CHB, 100 CHS and 89 JPT) [26] to infer *Std* and *Inv* haplotypes. In this case, there are two main challenges for SNP phasing. First, the inversion has a low frequency and there are no *Inv*/*Inv* homozygotes in the 1000GP Phase 1 dataset from where to recognize SNP variants present exclusively in the derived arrangement (which are always found in *Std*/*Inv* individuals and are difficult to place in one of the chromosomes unequivocally). Second, inversion length increases the possibility of phasing errors [43]. To minimize these effects, we used the BEAGLE software [44] to filter and call the SNP genotypes based on their likelihoods in each individual, and then to phase the different variants (see Methods for details). A total of 15,799 high-quality SNPs in 570 phased haplotypes (excluding the two chromosomes of one heterozygote individual, in which a phasing error between inversion breakpoints was detected) were finally included in posterior analyses.

These haplotypes were used to estimate the proportion of each chromosome that belongs to a number K of hypothetical ancestral populations with the software STRUCTURE [45]. Notably, the *Inv* arrangement shows a single component even when a high number of ancestry components (K = 10) is considered (S5 Fig). At the well-supported value of K = 5, the proportion of the *Inv* component in *Std* chromosomes is 12.9% in JPT, 6.3% in CHB and 10.5% in CHS, which suggests that *Inv* haplotypes are similarly related to any group of *Std* chromosomes and probably appeared before the establishment of the different East Asian populations, as the estimated age suggests. In addition, this highlights the highly homogeneous composition of *Inv* haplotypes due to its relatively recent and unique origin as well as the lack of gene flow between arrangements. Similar results were obtained by building an haplotype network, in which all the haplotypes with the inversion form a closely related and monophyletic group (S5 Fig).

**Fig 5:**
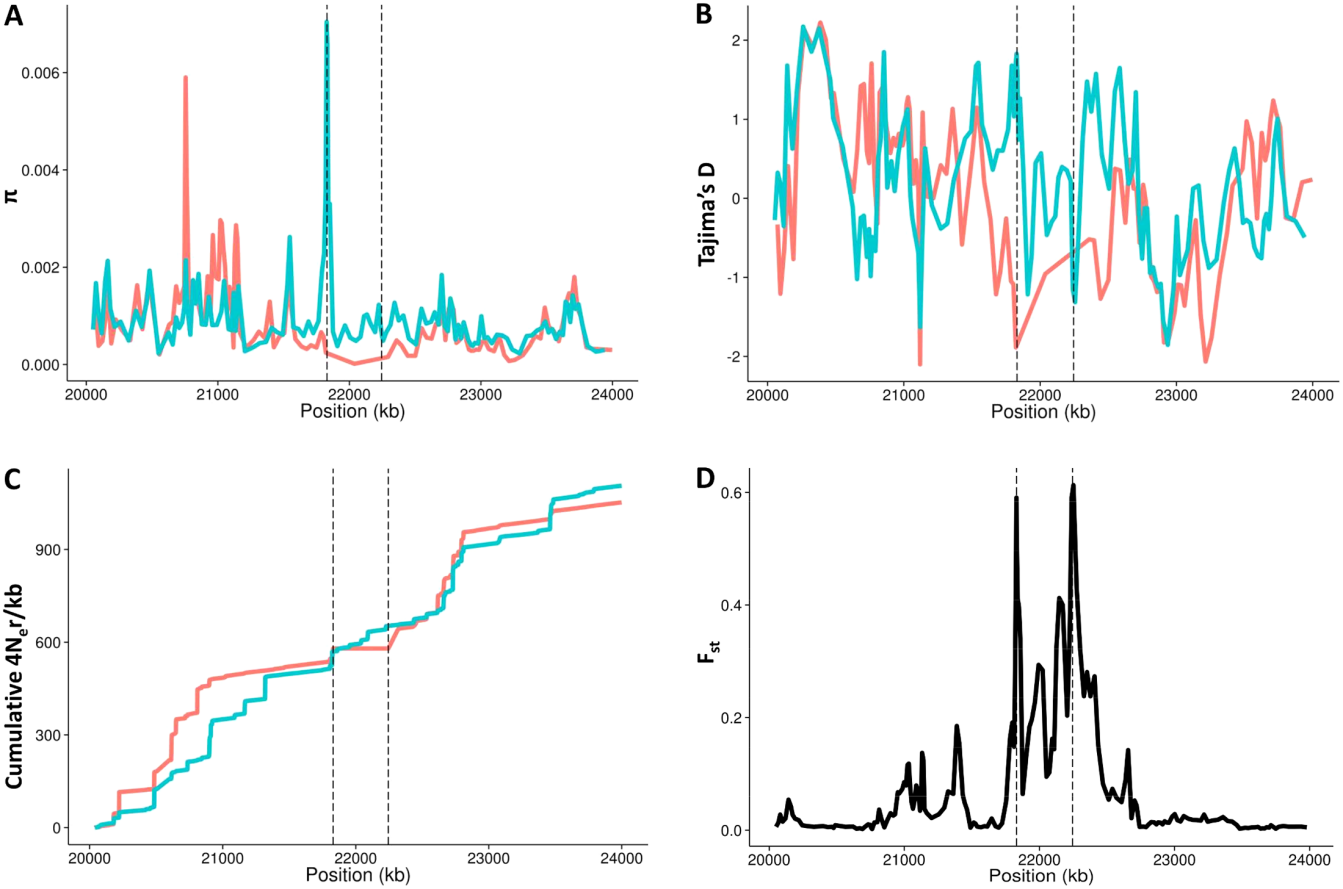
Recombination and nucleotide diversity patterns in the inversion region. **A.** Nucleotide diversity (π). **B.** Tajima’s D test statistic. **C.** Cumulative genetic length in 4N_e_r units. **D.** F_st_ values between *Std* and *Inv* chromosomes. HG19 assembly coordinates are shown in the X-axis, including the inverted region (marked by vertical dashed lines) and 1.8 Mb of flanking sequence at each side. Blue lines correspond to East Asian *Std* chromosomes and red lines to *Inv* chromosomes. All values were estimated in non-overlapping windows of 80 SNPs each considering the 285 phased East Asian individuals, except the recombination rate that was calculated between consecutive SNPs in the 24 *Inv* chromosomes and 24 random East Asian *Std* chromosomes. Tajima’s D statistic was Z-transformed to counteract the loss of low frequency SNPs during the phasing process.

Although phasing and SNP imputation errors might affect nucleotide variation estimates, we have also examined the global variation patterns of π and Tajima's D statistic [46] along and beyond the inverted region in the 1000GP Phase 1 dataset (Fig 5). Consistent with the age estimates, there are low levels of nucleotide diversity and negative values of Tajima's D within the inverted haplotypes, which extend outside the breakpoints. We then tried to determine the effects of the inversion on recombination by inferring population-scaled recombination rates (4N_e_r/kb) between each pair of consecutive SNPs in the 24 *Inv* and 24 randomly sampled *Std* phased chromosomes from the three East Asian populations. As expected since most *Inv* chromosomes are found in heterozygotes, there is a marked reduction in recombination within the inverted segment (Fig 5), which shows a genetic length of 83 4N_e_r units in *Std* East Asian chromosomes and only 5 in *Inv*. Accordingly, the distribution of F_st_ values between SNPs in *Std* and *Inv* chromosomes shows increased differentiation within the inverted region, which is maximal at the breakpoints (Fig 5). This reduction of recombination in heterozygotes is apparent even taking into account that the phased chromosomes used in this analysis can include a higher proportion of shared variants due to the phasing problems explained above. In fact, in the high-coverage SSMP data only two SNPs with enough read support were found to be shared between arrangements and were likely generated by gene conversion.

**Fig 6:**
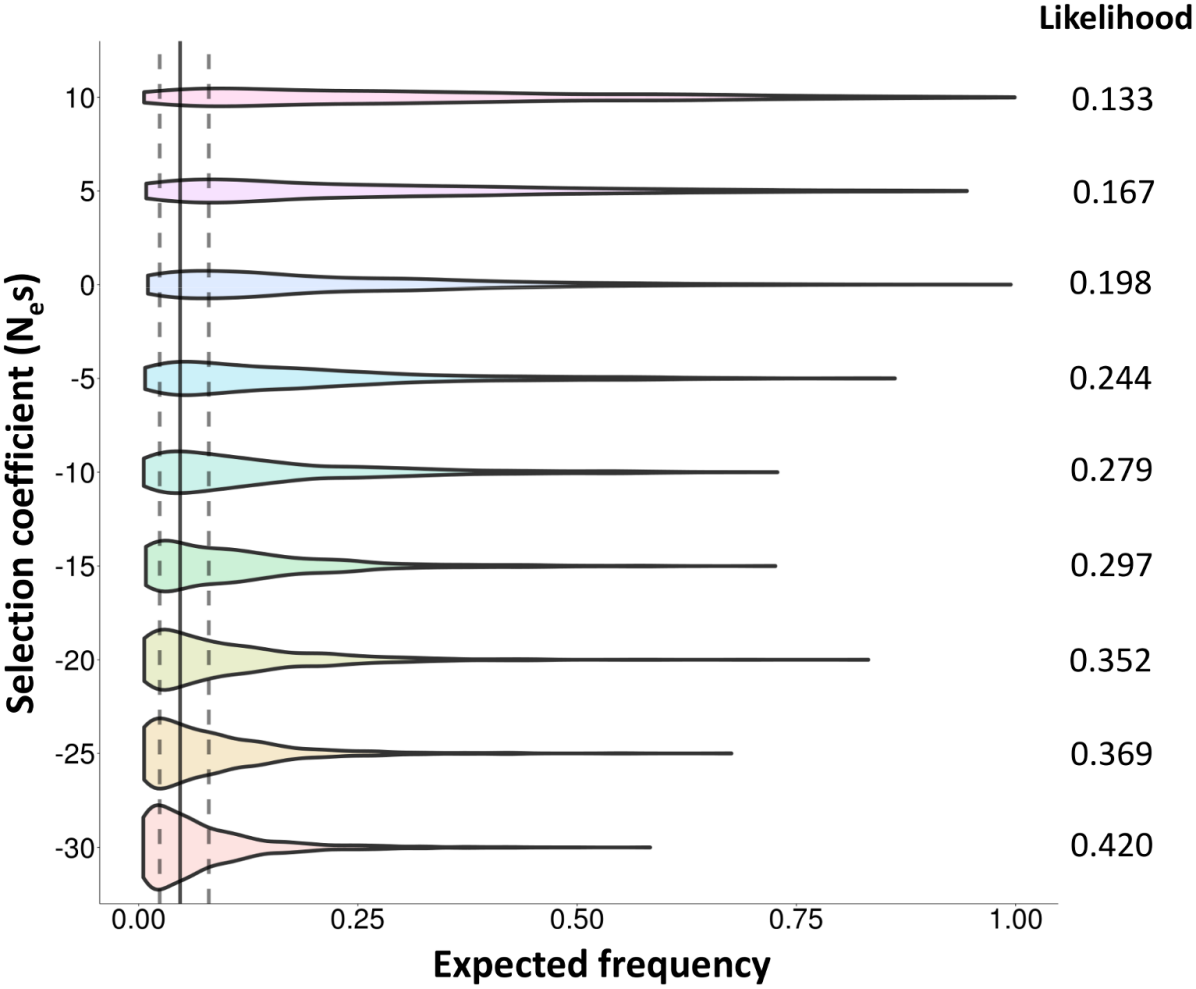
Evolutionary history of HsInv0379 inversion. Distribution of expected frequencies for a mutation arising 43,450 years ago under a model of human demography and different evolutionary scenarios according to forward-in-time simulations. Violin plots show the allele frequencies in 1,000 simulations for each selection coefficient (N_e_s between -30 and +10). The vertical solid line corresponds to the average frequency of the inversion in East Asia (4.73%) and dotted lines mark the range of frequencies observed in actual East Asian populations (2.4–8%). The likelihood of each N_e_s value (probability to obtain the observed frequencies) is shown at the right of the graph.

Finally, we looked for evidences of natural selection acting on this inversion with two approaches. First, we searched for recent partial selective sweeps in this region by applying the integrated haplotype score (iHS) test [47] to the 5,890 SNPs with available ancestral state information (37.3% of the phased SNPs). Although this test may be affected by recombination inhibition, no significant signals of recent positive selection were detected for the proximal (*P* = 0.67) or distal (*P* = 0.83) breakpoints or any SNPs in high LD with the inversion. Second, we used forward-in-time simulations to compare the observed frequency of the inversion with that expected under a human demographic model [48,49] and different evolutionary scenarios (Fig 6). Population-scaled selection coefficient (N_e_s) values corresponding to positive (+10 and +5) and purifying selection (-5, -10, -15, -20, -25 and -30) acting on a mutation of ~43,450 years were considered, together with a neutral change (N_e_s = 0). The results show that the probabilities to observe the actual frequencies (2.4–8%) in positive selection simulations are lower than those from a neutral mutation (Fig 6). On the other hand, these probabilities are higher with increasingly negative selection coefficients (Fig 6), which suggests that the inversion could be evolving under purifying selection and therefore that it may have negative consequences for its carriers. However, the likelihoods of negative selection coefficients are not significantly different from that of a neutral allele according to the log-likelihood ratio test, and the possibility that the inversion is neutral cannot be ruled out.

## Discussion

In this work we analyze in detail a new human polymorphic inversion (HsInv0379) that is the first with a clear effect on genes. Unlike other previously described long human polymorphic inversions that are mediated by large and complex repeats [29,50], in the present variant breakpoints are very simple, without repeats or even microhomology sequences, which suggests that it was probably generated by non-homologous mechanisms [30,51,52] and has a unique origin. After genotyping 2,667 independent individuals from 27 worldwide populations using three methods (PCR across breakpoints, reads spanning inversion breakpoints, and tag SNPs), we have determined that this inversion is almost exclusively found in East Asian populations with an average frequency of 4.7%, although frequency varies between 2.4 and 8% with populations located at the southern part of China exhibiting a higher frequency of the *Inv* allele.

Although the low frequency of the inversion complicates age calculations, our estimates using high-coverage sequencing data from a single population indicate that the inversion is 40,000–50,000 years old, which is relatively young compared to other human polymorphic inversions [29,53]. Accordingly, we propose that this inversion was generated from the ancestral *Std* chromosome after the out-of-Africa event around the time of the split between Asian and European populations 20,000–40,000 years ago [54,55], which agrees well with its distribution across all the analyzed East Asian populations as well as in one Bengali individual [56]. The finding of the *Inv* tag SNPs in a single Mexican individual whose genome does not have any component indicative of a recent ancestor of Asian origin [57], is consistent with the inversion predating the migration into the Americas ~15,000–23,000 years ago [54,58] and opens the possibility that the inversion might be present at low frequency in some American populations as well. The low nucleotide variation within *Inv* chromosomes with only a few polymorphisms specific of this arrangement also supports a recent origin for inverted haplotypes, which have not had time to accumulate much variation.

Inversions are known to affect phenotype in many species [4–7,9], but the molecular mechanisms underlying these effects remain unclear [3]. In the case of HsInv0379, no expression differences have been detected for any of the genes captured within the inverted segment, but one of the inversion breakpoints completely disrupts a gene, thus raising the possibility of phenotypic consequences derived from this mutational effect. The decrease of *ZNF257* expression level in inversion carriers and complete absence in the *Inv/Inv* individual indicate that this gene is not being expressed from the *Inv* chromosomes, although there is some degree of variation in the expression of the gene in *Std* chromosomes. In particular, variability is high among *Std*/*Std* individuals in available RNA-Seq data (S3 Fig), but it is not significantly different from that of other genes with low expression levels, which complicate their accurate quantification. However, despite the existence of other regulatory genetic variants, we have seen that the inversion is the main determinant of *ZNF257* expression in the carriers (S3 Table).

*ZNF257* belongs to the largest family of human transcription factors (KRAB-zinc finger proteins) [59] and it is positioned within a cluster of many similar genes. KRAB-zinc finger proteins are able to interact with chromatin-remodeling factors to repress transcription and have been involved in processes as diverse as development, differentiation, metabolism, apoptosis or cancer [59]. *ZNF257* was first isolated in bone marrow [60] and appears to be expressed in multiple tissues, mainly in testis, LCLs, ovary and thyroid (according to RNA-Seq data on different human tissues from both GTEx [37] and Illumina Body Map (http://www.ebi.ac.uk/gxa/experiments/E-MTAB-513). The type of cellular process it regulates or any target genes under its control are not yet known, although different evidences point towards a non-essential role of this gene. Based on exome data, *ZNF257* has been classified as a gene tolerant to functional genetic variation [61] and it shows loss-of-function mutations in heterozygosis in two individuals out of 1,092 in the 1000GP Phase 1 dataset. In addition, as expected by the inversion frequency, we have detected two *Inv*/*Inv* homozygotes where this gene is completely inactivated. Also, *ZNF257* is conserved in non-human primates, where it appears to be under purifying selection (dN/dS = 0.5443 with chimpanzee), but does not present a one-to-one ortholog in other mammal species. Here we provide support for an association between *ZNF257* down-regulation and expression changes in other genes in *trans*: (1) Expression differences in the same direction are observed independently using a different technique and a larger number of samples; and (2) *Std*/*Std* individuals with low *ZNF257* expression show expression levels similar to those of inversion carriers in at least two of the genes tested by qPCR (and in both cases the genes are up-regulated in inversion carriers, which is consistent with a lower amount of a transcriptional repressor like ZNF257). It is important to note that the detection of expression changes in *trans* is complicated by several facts: (1) many factors can determine the level of expression of a given gene and the inversion just alters the levels of one transcription factor; (2) the effects of *ZNF257* disruption in heterozygotes might be moderate due to buffering by *ZNF257* functional copy and they are likely to be larger in *Inv*/*Inv* individuals; and (3) lymphoblasts may not be the tissue where *ZNF257* expression reduction has its main effects. Still, this represents the best approach available to detect the functional consequences of this polymorphic inversion.

Biological relevance of *ZNF257* decrease due to the inversion may be questioned in light of the range of expression levels detected for this gene in *Std/Std* individuals (S2 and S3 Figs). However, gene disruption is not the only mechanism by which this inversion may be affecting gene expression and having potential phenotypic consequences, since it also generates a fusion transcript at BP1. In fact, at least one of the differentially expressed genes validated by qPCR (*CACNB2*) showed a level of expression in *Std/Std* individuals with low *ZNF257* closer to that in the other *Std*/*Std* individuals than to inversion carriers, suggesting that the expression change is associated with the inversion itself, rather than with *ZNF257* decrease, and the fusion transcript could be involved. This transcript has no homology to known transcripts, but coding of short peptides [62] or binding to proteins or microRNAs cannot be ruled out [63,64]. The new exon is entirely transcribed from TE sequences, but it contains the sense strand for both *Alu* and *L1* fragments and it should not bind RNAs from other TE copies. The higher expression level compared to *ZNF257* when both are driven by the same promoter suggests that the fusion transcript is taking advantage of new regulatory elements in its new position or operating without the influence of negative regulators, supporting the effect of chromosomal position on gene regulation [16], although post-transcriptional mechanisms could play a role as well. Nevertheless, additional knockdown or overexpression experiments would be needed to really show a causal link between *ZNF257* and the fusion gene to other gene-expression changes.

Although we tried to establish functional relationships among the differentially expressed genes in inversion carriers, no clear significant categories emerged that could indicate the cellular processes that may be affected. However, there are several genes that may have important functions in the cell (S5 Table), like *BCL11A* that acts as a switch between fetal and adult hemoglobin in erythrocytes, is essential for B-lymphocyte development [65] and has recently been involved in autism [66], or *APP*, a protein involved in autosomal dominant Alzheimer’s disease [67]. Moreover, several non-coding RNAs of unknown function show quite high fold changes in inversion carriers (S5 Table), such as the processed pseudogene RP11-343H5.4 (8.7-fold increase), or the lincRNA *SNHG5* (4.0-fold increase), whose introns produce snoRNAs and is located at the breakpoint of a chromosomal translocation involved in B-cell lymphoma [68].

Assuming that any moderately long inversion should have some negative consequences due to the generation of unbalanced gametes by recombination in heterozygotes, the mutational effects at the breakpoints could result in several possible evolutionary scenarios. The inversion could behave as positively selected, neutral or nearly neutral depending on whether the potential beneficial effects of the *ZNF257* disruption, fusion transcript and the associated expression changes overcome the negative fertility effects or if the fertility effects are very small. On the other hand, if both effects are negative, the inversion could range from slightly to strongly deleterious. Thus, we have simulated the evolution of a mutation arising ~43,000 years ago under a human demography model with neutrality and different strengths of positive and purifying selection. While the observed inversion frequencies are compatible with both neutrality and negative selection and the age uncertainty might alter the simulation outcomes, the actual values are more likely under the assumption that the inversion is a deleterious polymorphism that cannot rise in frequency in the population due to its negative effects.

A human individual typically carries ~20 genes completely inactivated, but in more than ¼ of these only some transcripts are affected [69]. Loss-of-function mutations tend to occur in genes that are part of a family and less evolutionary conserved and this type of changes show low frequencies, all of which fit perfectly our situation (although according to a recent study in the Icelandic population [70], 85% of loss-of-function mutations have a frequency below 0.5%, which is much lower than the 4.7% detected for the present inversion in East Asians). However, many genes involved in late-onset diseases will behave as neutral variants from an evolutionary point of view, but still may have functional consequences able to impact human health [71]. Alternatively, if we consider that recombination is not physically inhibited in the inverted region in heterozygotes, we can use its genetic length in *Std* chromosomes, 83 (in 4N_e_r units), as a proxy for the population-scaled selection coefficient (4N_e_s) acting on the inversion associated to the probability of harboring crossovers within the inverted sequence between *Std* and *Inv* chromosomes, which results in a N_e_s ≈ -21. Since similar (or even higher) negative coefficients seem to be the parameters that best fit the observed inversion frequencies, both mechanisms (recombination and gene disruption) may have a role in causing the negative effects of this inversion. In that case, gene surfing during the expansion of humans in Asia [72] could help to explain the observed frequencies of such a deleterious variant. So far we have been unable to detect an association of the inversion with disease markers and other phenotypic traits from blood tests in a Japanese cohort. Nevertheless, future association analysis with a higher number of individuals to overcome the reduced power to detect significant effects for low-frequency variants like HsInv0379 (just 1.95% in the analyzed population) and a more complete battery of phenotypes tested, should contribute to elucidate the possible functional consequences of this inversion.

### Ethics statement

Procedures that involved the use of human samples were approved by the Animal and Human Experimentation Ethics Committee (CEEAH) of the Universitat Autònoma de Barcelona (Ref. 821H). Protocols for the Nagahama cohort project were approved by the Kyoto University Graduate School and Faculty of Medicine Ethics Committee (Ref. G278) and written informed consent was obtained from all of the participants.

## Materials and Methods

### Samples and DNA isolation

A total of 547 samples from eight populations of HapMap and 1000GP [26,73] projects have been used (see Table 1 for details). Ninety unrelated individuals were analyzed in each of these populations except for CHB (49) and JPT (47) plus a single CDX individual, and the CEU and YRI populations, which are organized in 30 parent-children trios. Genomic DNAs were obtained from Coriell Cell Repository (Camden, NJ, USA), except most of those from the CEU population and those of the samples used for the expression analysis, which were isolated from LCLs (Coriell Cell Repository, Camden, NJ, USA) as previously described [74] or using the PrepFiler Forensic DNA Extraction kit (Life Technologies). Fosmid ABC9-45236600J18 was obtained from Evan E. Eichler (University of Washington, WA, Seattle) and DNA was isolated from 1.5 ml of overnight bacterial TB culture following a typical alkaline lysis plasmid DNA extraction protocol [75].

### PCR/RT-PCR amplification

For inversion genotyping, one primer in region C within the inverted segment was used in combination with primers in regions A and D outside the inversion (S7 Table) to generate products AC (*Inv*) and/or CD (*Std*). PCR amplifications were performed in a reaction volume of 25 μl including 1x buffer, 1.5 mM MgCl_2_, 200 μM each dNTP, 0.4 μM each primer, 1.5 U Taq DNA polymerase (Roche) and 50–100 ng genomic DNA. Typical cycling conditions were 2 min at 94°C of denaturation followed by 35 cycles of 30 sec at 94°C, 30 sec at 60–65°C and 30–60 sec at 72°C, and a final step of 7 min at 72°C. In long-range PCRs, the same reaction conditions were used except for 240 μM each dNTP, 2.5 U Pfu Turbo DNA Polymerase (Stratagene) and 1–10 ng of fosmid DNA, together with cycling steps of denaturation at 95°C and extension at 68°C for 9 min. For RT-PCR, total RNA from 27 East Asian LCLs (Coriell Cell Repository, Camden, NJ, USA) was isolated using Trizol (Life Technologies) and cDNA synthesis was carried out after DNase treatment (DNA-free, Ambion) using SuperScript First Strand Synthesis System for RT-PCR (Invitrogen) from 1 μg total RNA with a combination of oligodT and random primers. Controls without retrotranscriptase were performed for all samples to exclude DNA contamination. RT-PCR reactions were carried out in the same conditions described above with 1 μl of cDNA as template. When needed, PCR products were sequenced by the Sanger method at Macrogen (Seoul, Korea).

### RNA-Seq and differential expression analysis

Total RNA was isolated from 15-ml cell cultures of eight East Asian LCLs collected before reaching saturation (4.5–8.6 × 10^6^ cells), using miRNeasy Mini kit with on-column DNase digestion (Qiagen). RNA quality and quantity were measured by absorbance with Nanodrop (Thermo Scientific), fluorescence on a Qubit instrument (Life Technologies) and 2100 Bioanalyzer (Agilent Technologies). TruSeq cDNA libraries (Illumina) were prepared from 500 ng total RNA with polyA capture, and 2 × 100 bp paired-end sequencing was performed on the Illumina HiSeq 2000 platform at Beckman Coulter Genomics (Danvers, MA, USA). A total of 413 million single reads were produced (51.7 million per sample on average; S4 Table). We mapped fastq files of each sample against the GRCh37 (H19) human genome with TopHat2 (default parameters), adding the Ensembl release 73 (Gencode v18) gene annotation to guide the alignments, and HTSeq [76] was ran to count the number of reads for each gene based on the same gene annotation. To detect differentially expressed genes, we applied DESeq2 [77] and selected genes with FDR < 0.1. Protein coding genes and non-coding genes were analyzed separately. Permutations and simulations were carried out as detailed in S4 Fig. Functional relationships among differentially expressed genes (FDR < 0.05) were analyzed with the DAVID Functional Annotation tool [78]. Chimeric transcripts originated across the inversion breakpoints were detected with the IdeGen pipeline [79] using the gene annotation file (Ensembl 73, Gencode v18), inverted region sequence in GenBank format, human genome sequence (HG19) and the DESeq2 normalized fastq files for each sample. The sequence of the inverted region was created *in silico* by inverting the segment between breakpoints and adding 1 Mb of flanking region at each side.

### Quantitative PCR (qPCR)

In quantitative real-time RT-PCR, 20-μl reactions were carried out in an ABI Prism 7500 Real-Time PCR System (Applied Biosystems) with iTaq SYBR Green Supermix with Rox (Bio-Rad), 75–125 nM of each primer (S7 Table) and 1 μl of a 1/10 dilution of the cDNA sample (except for gene *ZNF257* that was amplified from 0.5 μl of undiluted cDNA due to its low expression levels). An independent RNA sample isolated from a different cell culture was used for those individuals previously analyzed by RNA-Seq. All samples were measured in triplicate by relative quantification with a standard curve and a final dissociation curve stage. Genes *POLR2F* and *ACTB* were amplified as reference genes to control for differences in cDNA concentration. Mean expression values for each genotype group (*Std*/*Std vs*. *Std*/*Inv*) were compared with a Student’s t-test.

### *In silico* inversion genotyping

To search the *Std* and *Inv* sequences at inversion HsInv0379 breakpoints among the individuals sequenced by the 1000GP [26], we prepared a fasta file with the 100 bases surrounding the two breakpoints (S2 Table). If the 1000GP reads had already been aligned to the reference genome, only unmapped reads and reads mapped to the breakpoint regions were downloaded from the ftp server in SAM format with SAMtools [80], and then converted to fastq. Otherwise, the raw fastq files were downloaded, avoiding color-space sequences and exome reads. The reads were processed with a slightly modified version of BreakSeq [52] as described previously [81]. Reads overlapping at least 10 bases of either side of a breakpoint were retained (regardless of their length), and only those mapping uniquely to an *Inv* or *Std* breakpoint with no other hits in the reference genome were counted as allele observations. A total of 1,892 individuals from the 1000GP [26] were analyzed sequentially between January and August 2013. For inversion genotyping with the three tag SNPs, SNP calls were obtained from the 1000GP Phase 3 vcf files (20130502 release). For those individuals with alternative alleles, allele count was checked using mpileup function of SAMtools v 0.1.19 [80] with default parameters (minimum base quality Q = 13).

### Nucleotide variation analysis

LD between the inversion and SNPs in the inverted region plus 20 kb of flanking sequence at each side from the 1000GP Phase 1 SNP data [26] was calculated with Haploview v4.1 [82] (both for each population separately and the seven HapMap populations together), using unrelated individuals for which the inversion had been genotyped by PCR. Only SNPs in perfect LD with the inversion (*r* ^*2*^ = 1) were considered tag SNPs. Next, haplotypes were inferred using BEAGLE package v3.3.2 [44] from 1000GP Phase 1 SNP data [26] in the inverted region and 1.8 Mb of flanking sequence at each side for 286 East Asian individuals. Both inversion breakpoints were included in their corresponding genomic positions as extra variants with two alleles (*Std* and *Inv*) to assess the presence of switch errors in phasing. Two different phasing strategies were used. The first phasing run was carried out with default (fast) parameters including all 1000GP SNPs but using genotype likelihoods to call SNP genotypes. The second one was aimed to increase accuracy by excluding all markers with allelic *R* ^*2*^ < 0.9 in the first run (larger values of allelic *R* ^*2*^ indicate more accurate genotype imputation), and increasing to 20 both the number of iterations of the phasing algorithm and the number of sampled haplotype pairs for each individual during each iteration. In the accurate phasing, a switch error between breakpoints was detected in only one inversion carrier (compared to only two inverted chromosomes correctly retrieved using the first phasing run).

To determine the genetic substructure within the inverted region and the likely number of ancestral population groups (K) of the 570 phased East Asian chromosomes, we used a Bayesian genetic clustering algorithm (STRUCTURE v2.3) [45]. We assumed the admixture model and ran 100,000 Markov chain Monte Carlo iterations with a burn-in period of 100,000. We also ran STRUCTURE assuming the linkage model, and we obtained equivalent results. We did not use prior population information and K values between 1 and 10 were tested to estimate their posterior probability. Estimates of recombination rate were obtained using the *rhomap* program distributed within the LDHat package v2.2 [83] and the same strategy used in Alves *et al*. 2014 [84]. For each pair of adjacent SNPs we obtained five estimates of the population recombination rate (*p* = *4N*_*e*_ *r*/kb) and the median was used in the analysis. Recombination was estimated separately in a random sample of 24 *Std* phased chromosomes and the 24 phased *Inv* chromosomes. π [85] and Tajima's D [46] statistics were estimated at non-overlapping windows of 80 SNPs. The alteration of the site frequency spectrum produced by the phasing algorithm has a minor effect on recombination rate estimates or population structure inference, but it could have a big effect on polymorphism level estimates. Thus, the Tajima's D distribution was Z-transformed and centered at 0 to try to correct for the systematic overestimation caused by the preferential filtering of low frequency variants during the phasing procedure.

### Inversion age estimation

A divergence-based estimate of the inversion age [42] was calculated using high-coverage SNP data from the SSMP [35]. Nucleotide variation was analyzed in the inverted region (chr19: 22245260–21863433), excluding the segmental duplication copy at BP1 (chr19:22245260–21863433), which shows an increased nucleotide diversity that may alter final results (Fig 5), and 2.3% of SNPs with missing values for some individuals. We assumed that variants in heterozygotes found also in *Std* homozygotes belonged to the *Std* chromosome, while those variants found exclusively in more than one heterozygote had appeared in *Inv* chromosomes. A proportion of the singletons found in heterozygotes were assigned to *Std* chromosomes based on the observed singleton frequency in *Std* homozygotes, and the rest were attributed to *Inv* chromosomes. We calculated the net pairwise nucleotide differences between orientations (*d*_*A*_) by subtracting nucleotide diversity observed in *Std* sequences (π_*S*_) to the total nucleotide differences between *Std* and *Inv* sequences (*d*_*S-I*_) (equations 10.20, 10.21 and 10.22 in Nei 1987 [85]). The divergence time between *Std* and *Inv* chromosomes was estimated with the formula *T* = *d*_*A*_ */2s* (where *T* is time and *s* is the local substitution rate). The 95% confidence intervals for age estimates were obtained with 1,000 bootstrap resamplings of 96 individuals. The local substitution rate was estimated with the same formulas as before using chimpanzee as outgroup and assuming a divergence time with humans of 5–6 Mya. Alignment between human (HG19) and chimpanzee reference genomes (panTro4/CHIMP2.1.4) was retrieved from Ensembl database.

### Detection of natural selection

The integrated haplotype score (iHS) test [47] was performed on those SNPs located within the inverted region ± 1.8 Mb with known ancestral state (5,890 SNPs). To determine if the inversion itself is undergoing positive selection we compared the standardized iHS value for the breakpoints to the overall iHS distribution. To further check the role of linked selection on inversion current frequency, LD with the inversion was calculated for the list of SNPs with significant iHS values (*P* < 0.05). The expected frequency distribution of hypothetical mutations with different selective effects that appeared ~43,450 ya under the human demographic history inferred by Gravel *et al*. 2011 [48] was estimated by forward-in-time simulations. The demographic history included an ancient African expansion (~177,000 ya), an out-of-Africa bottleneck (~62,000 ya), a founding of Asia bottleneck (~28,000 ya), an initial phase of exponential growth within Asia, and a recent explosive growth phase (starting ~5,000 ya). All simulations were performed using SFS_CODE [86] and the same parameters as in Maher *et al*. 2012 [87] were used except for the founding-of-Asia bottleneck which is more severe. Simulations consisted in 10^6^ iterations of a gene with length 1 bp, initial population of 2,000 diploid individuals, random mating between males and females, without mutation in each generation, and sampling at the end 96 diploid individuals (from the terminal population size of 150,408 individuals) to calculate allele frequency. All extinct or fixed iterations were discarded and only the first 1,000 iterations still segregating were kept for analysis. Years were converted to generations by rescaling to the ancestral population size of 7,310 [48] and assuming 30 years per generation. The command line for SFS_CODE used here is "sfs_code 1 1000000-A-n 96-N 2000-P 2-t 0-L 1 2-I-r 0-W 0-Td 0 1.9800273598-Td 0.2211582307 0.1285753765-Tg 0.28499772 44.75089-Td 0.28499772 0.2976894143-Tg 0.3260373917 282.7192-TE 0.3374373005—mutation 0.238372093 S 0 G <selection coefficient>". Parameter likelihood was estimated as the probability to find the 2.4–8% observed frequency values in East Asian populations in each simulated scenario and selection coefficients were compared to the neutral model (N_e_s = 0) using a likelihood ratio test.

### Phenotypic trait association

Inversion tag SNP rs148316037 was genotyped by TaqMan assay (Applied Biosystems) (S7 Table) from 5–10 ng of DNA of 3,824 Japanese subjects from the Nagahama Prospective Genome Cohort for Comprehensive Human Bioscience (The Nagahama Study), a community-based prospective multi-omics cohort study conducted by Kyoto University in Japan [39]. Complete linkage between the inversion and rs148316037 in this cohort was confirmed by genotyping the inversion by PCR in a total of 56 individuals (33 *Std*/*Std*, 22 *Std*/*Inv* and 1 *Inv*/*Inv*). We performed quantitative linear regression and logistic linear regression to analyze the associations between genotypes of rs148316037 tag SNP as a proxy for the inversion and clinical phenotypes (S6 Table). The quantitative traits were transformed by rank-based inverse normalization method to fit normality and used as dependent variables in the linear regression analyses. Age and sex were used as covariates.

### Data availability

All the data described here have been deposited at the InvFEST Human Polymorphic Inversion Database (http://invfestdb.uab.cat/report.php?n=HsInv0379). DNA sequences have been deposited at GenBank (accession numbers KT592300-KT592304). RNA-Seq data are available at the Sequence Read Archive (SRA290330).

## Supporting Information

S1 Fig Breakpoint sequence analysis and identification. **A.** Breakpoint 1 (BP1) isolation from fosmid ABC9_45236600_J18. The fosmid 40-kb insert is represented by a black line (top) with sequenced segments shown below as grey bars. Primers designed within the innermost sequences mapping in distinct regions of the reference genome (A1 and C1) were used to amplify a 7-kb PCR product containing the inversion BP1. This PCR product (bottom) was digested with restriction enzymes *EcoR*I (E), *Pst*I (P) and *Hind*III (H) and by comparison with the expected restriction patterns from these regions in the reference genome, BP1 was localized within a 1.5 kb segment marked with a red box. Primers A4 and C5 were used to amplify and sequence this fragment. **B.** RepeatMasker results showing annotations of transposable element (TE) blocks of 14.3 kb at BP1 (top) and 4.9 kb at BP2 (bottom) in the reference genome (*Std*) and in the sequenced region of individual NA18956 inverted chromosome (*Inv*). LINE (dark green) and SINE (light green) fragments are depicted as arrows indicating TE orientation. The nucleotide identity between the two blocks is very low, with only several fragments of ~250 bp corresponding to *Alu* sequences showing 78–85% identity. TEs are interrupted exactly at BPs in *Inv* chromosomes, which suggests that *Std* is the ancestral orientation. **C.** Multiplex PCR assay used for inversion genotyping in seven HapMap populations (see Table 1). A heterozygote and a *Std*/*Std* homozygote are shown. PCR products and sizes are also indicated.
(TIF)

S2 Fig Individual expression levels of *ZNF257* and the fusion transcript. Normalized expression values for *ZNF257* (**A**) and the fusion transcript (**B**) obtained by qPCR are shown for 15 *Std*/*Std*, 11 *Std*/*Inv*, and 1 *Inv/Inv* individuals in different shades of blue from darkest to lightest. All expression values are given relative to sample NA18621 for *ZNF257* and NA18563 for the fusion transcript, which have an expression level of 1. *Std/Std* individuals with a low level of *ZNF257* expression are represented in red. Average expression levels for each inversion genotype are shown in Fig 3.
(TIF)

S3 Fig Variation of *ZNF257* expression in comparison with other genes expressed at different levels. Each dot represents the coefficient of variation (VC) of the expression of a gene in the Geuvadis RNA-Seq data [36] from the lymphoblastoid cell lines of 192 CEU, TSI and YRI individuals included in this study. Variation coefficient values are represented in function of the level of expression of the gene expressed here as the log2 value of the corresponding read count. *ZNF257* (red dot) is among the top 10% genes with an average log2(counts) less than 2.
(TIF)

S4 Fig Assessment of the reliability of the differential expression analysis. **A.** Differentially expressed genes detected in 18 permutations of the four *Std/Std* and *Std/Inv* samples with RNA-Seq data in which two individuals of each genotype group have been exchanged. False positive rate (FPR) indicates the proportion of genes identified between the resulting groups in each of the permutations, and the red line marks the proportion of differentially expressed genes (0.47%) in the comparison of the four individuals with each inversion genotype. Out of the 18 possible combinations, 14 show a lower number of genes compared to the groups determined by inversion genotype. The remaining four permutations all contain certain pairs of individuals in the two groups compared (NA18621 and NA18973 in one, and NA18632 and NA18951 in the other, where both pairs are formed by individuals with different sex, population, and inversion genotype) and might reflect some difference between these pairs of samples not taken into account. **B.** Histogram of the number of de-regulated genes in 400 simulations generated with the *rnbinom* function in R to simulate a negative binomial distribution of the read counts for eight samples with the mean and dispersion of the real data calculated by DESeq2. The red line shows the corresponding number of differentially expressed genes between the *Std/Std* and *Std/Inv* analyzed by RNA-Seq, which according to the results of the simulations has a p-value (*P*) of 0.055. In both the permutations and the simulations the analysis was done with DESeq2 using the same parameters and criteria than in the analysis of real data of S5 Table (FDR < 0.1), with the exception that the sex chromosomes were excluded and only coding genes were taken into account.
(TIF)

S5 Fig Genetic structure of the inverted region in East Asian populations. **A.** Proportion of each chromosome that belongs to different hypothetical ancestral populations. Each chromosome is represented as a vertical line colored according to the proportions of different ancestral components. The number of ancestral components (K) considered in each analysis is indicated at the left. A total of 570 phased chromosomes belonging to three East Asian populations from 1000GP Phase 1 are included. Populations to which *Std* chromosomes belong are shown below each graph. *Inv* chromosomes have a single distinctive component and group together although they come from the three analyzed populations. **B.** Median-Joining network from the same 570 phased chromosomes. Circles correspond to the different haplotypes found for the region of the inversion in the three East Asian populations with *Std* in different colors and *Inv* in blue. Circle sizes are proportional to the frequency of each haplotype and the branch length indicates approximately the number of mutations between them [74].
(TIF)

S1 Table HsInv0379 East Asian genotypes. PCR results, tag SNP genotypes and number of reads containing *Std* or *Inv* breakpoints are shown for each individual. Individuals shaded in red are related to other individuals in the list and were not considered for frequency calculations. Relationships were established according to family information available at Coriell Institute (https://catalog.coriell.org/) and 1000GP Phase 3 analysis (ftp://ftp.1000genomes.ebi.ac.uk/vol1/ftp/release/20130502/20140625_related_individuals.txt). SNP calls are those of the 1000GP Phase 3 or SSMP vcf files. Read support for each allele is also shown in inversion carriers except for the Malay population where data were not available. SNP alleles are always in the order Reference/Alternative that corresponds to *Std*/*Inv*. Discordant SNP genotypes among the three inversion tag SNPs are shaded in grey and an alternative allele call in two or more of the SNPs is required to be considered enough evidence of the inverted haplotype. The two non-Asian inversion carriers are also included. ND = not determined;— = not analyzed.
(XLSX)

S2 Table Breakpoint sequences used to detect reads spanning inversion breakpoints. Sequences located inside the inverted fragment are shown in red.
(XLSX)

S3 Table Effect of different genetic variants in *ZNF257* gene expression. *R* ^*2*^ represents the fraction of *ZNF257* gene expression variation in the qPCR analysis that can be explained by the inversion and several candidate eQTLs across either all the samples (*Std/Std*, *Std/Inv* and *Inv/Inv*) or *Std* homozygotes only assuming an additive model.
(XLSX)

S4 Table RNA-Seq summary information. Sample features and reads sequenced, mapped and analyzed in each individual. The gene annotation used is Ensembl release 73 (GENCODE v18).
(XLSX)

S5 Table Differentially expressed genes in the RNA-Seq analysis (FDR < 0.1). Genes in boldface were also tested by quantitative PCR (qPCR) and those in chromosome Y were excluded.
(XLSX)

S6 Table Results of the regression analysis between inversion genotypes and phenotypic traits in the Nagahama cohort. Linear regression has been used for continuous variables and logistic regression to test the association between the inversion and positivity for anti-CCP antibody, rheumatoid factor, antinuclear antibodies and their fluorescent patterns. Beta corresponds to the fitted regression coefficients (per allele) and SE to their standard errors. The phenotype with the lower *P*-value is indicated in italics.
(XLSX)

S7 Table Primers and probes used in this work. Primers were designed with the Primer3Plus program [88].
(XLSX)
