# Supplementary figures and images for: Functional Impact and Evolution of a Novel Human Polymorphic Inversion That Disrupts a Gene and Creates a Fusion Transcript
Source: PLoS Genet. 2015 Oct 1;11(10):e1005495. doi: 10.1371/journal.pgen.1005495 (PMC4591017; doi:10.1371/journal.pgen.1005495)

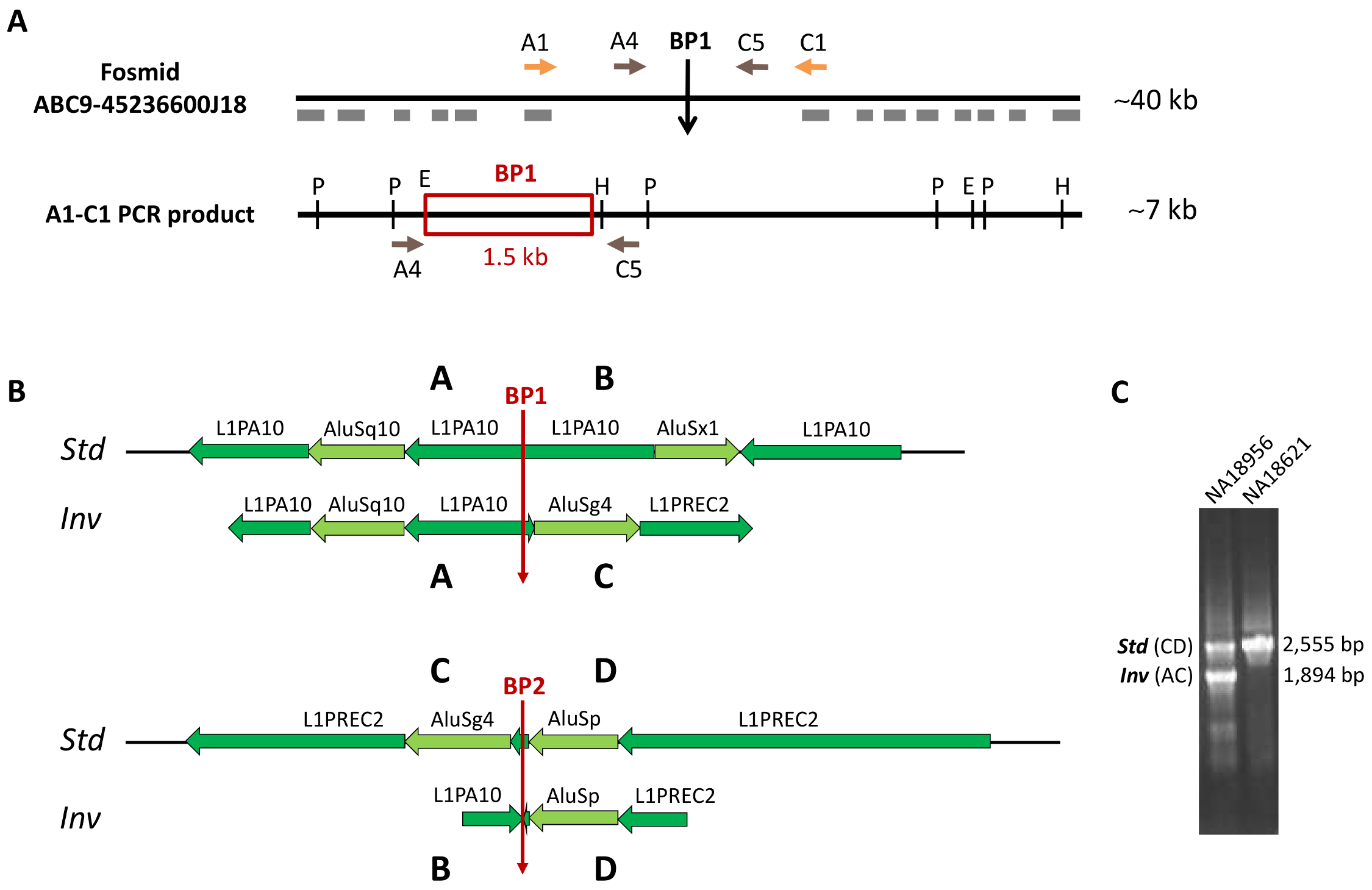

Supplement: S1 Fig — A. Breakpoint 1 (BP1) isolation from fosmid ABC9_45236600_J18. The fosmid 40-kb insert is represented by a black line (top) with sequenced segments shown below as grey bars. Primers designed within the innermost sequences mapping in distinct regions of the reference genome (A1 and C1) were used to amplify a 7-kb PCR product containing the inversion BP1. This PCR product (bottom) was digested with restriction enzymes EcoRI (E), PstI (P) and HindIII (H) and by comparison with the expected restriction patterns from these regions in the reference genome, BP1 was localized within a 1.5 kb segment marked with a red box. Primers A4 and C5 were used to amplify and sequence this fragment. B. RepeatMasker results showing annotations of transposable element (TE) blocks of 14.3 kb at BP1 (top) and 4.9 kb at BP2 (bottom) in the reference genome (Std) and in the sequenced region of individual NA18956 inverted chromosome (Inv). LINE (dark green) and SINE (light green) fragments are depicted as arrows indicating TE orientation. The nucleotide identity between the two blocks is very low, with only several fragments of ~250 bp corresponding to Alu sequences showing 78–85% identity. TEs are interrupted exactly at BPs in Inv chromosomes, which suggests that Std is the ancestral orientation. C. Multiplex PCR assay used for inversion genotyping in seven HapMap populations (see Table 1). A heterozygote and a Std/Std homozygote are shown. PCR products and sizes are also indicated. (TIF) [file pgen.1005495.s001.tif]

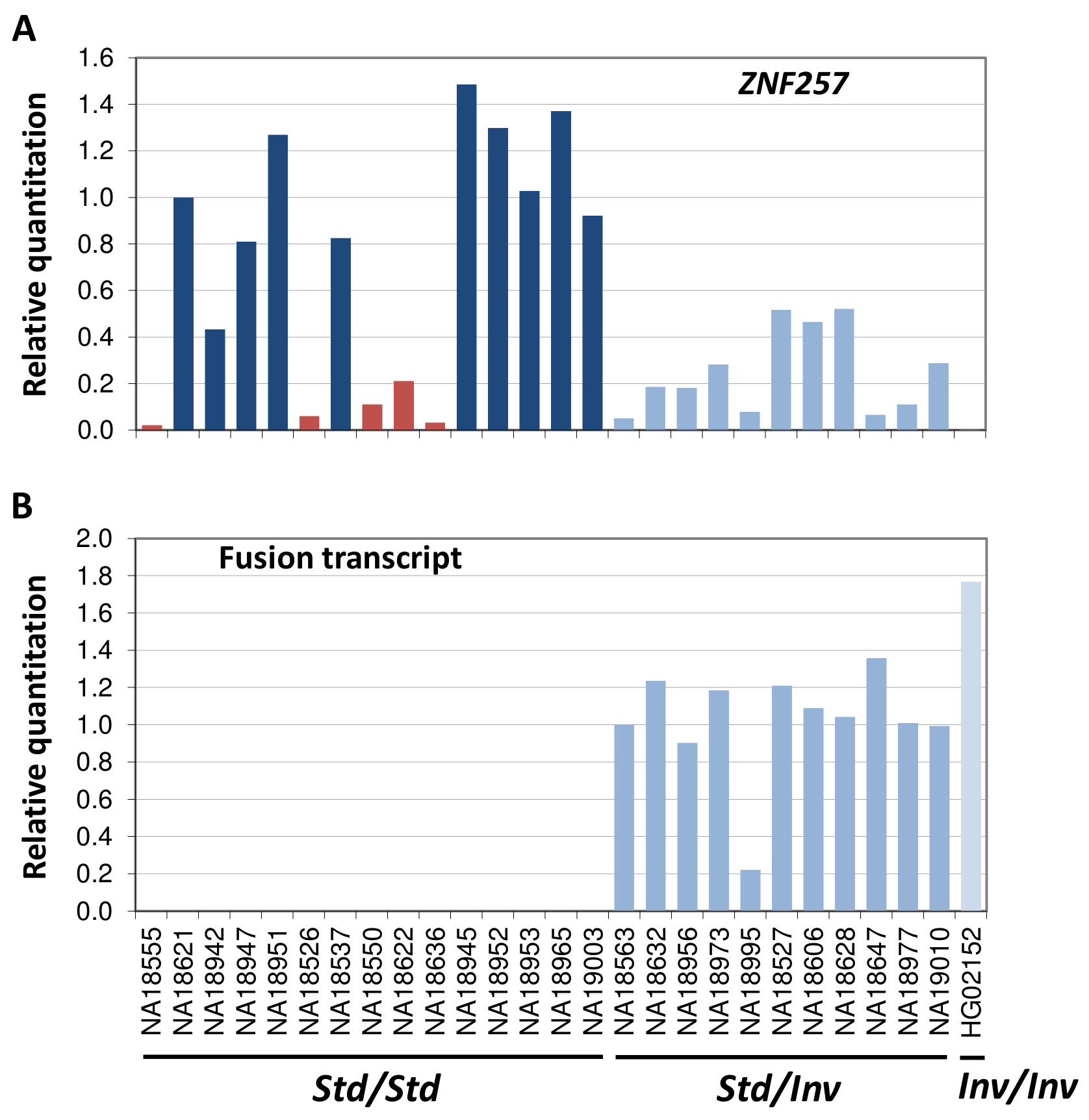

Supplement: S2 Fig — Normalized expression values for ZNF257 (A) and the fusion transcript (B) obtained by qPCR are shown for 15 Std/Std, 11 Std/Inv, and 1 Inv/Inv individuals in different shades of blue from darkest to lightest. All expression values are given relative to sample NA18621 for ZNF257 and NA18563 for the fusion transcript, which have an expression level of 1. Std/Std individuals with a low level of ZNF257 expression are represented in red. Average expression levels for each inversion genotype are shown in Fig 3. (TIF) [file pgen.1005495.s002.tif]

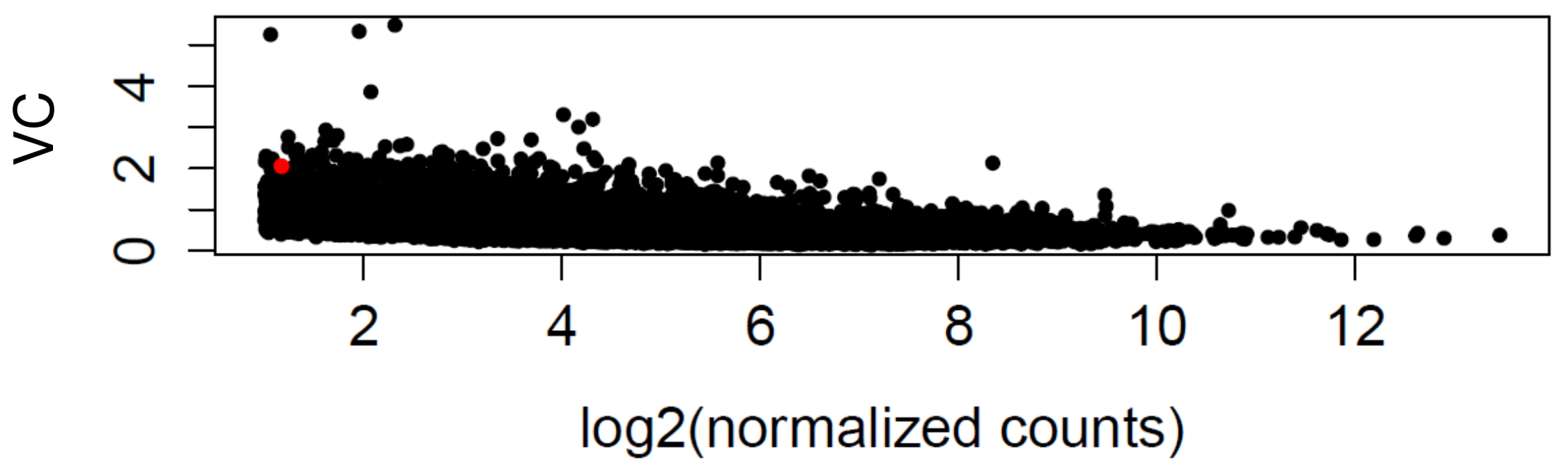

Supplement: S3 Fig — Each dot represents the coefficient of variation (VC) of the expression of a gene in the Geuvadis RNA-Seq data [36] from the lymphoblastoid cell lines of 192 CEU, TSI and YRI individuals included in this study. Variation coefficient values are represented in function of the level of expression of the gene expressed here as the log2 value of the corresponding read count. ZNF257 (red dot) is among the top 10% genes with an average log2(counts) less than 2. (TIF) [file pgen.1005495.s003.tif]

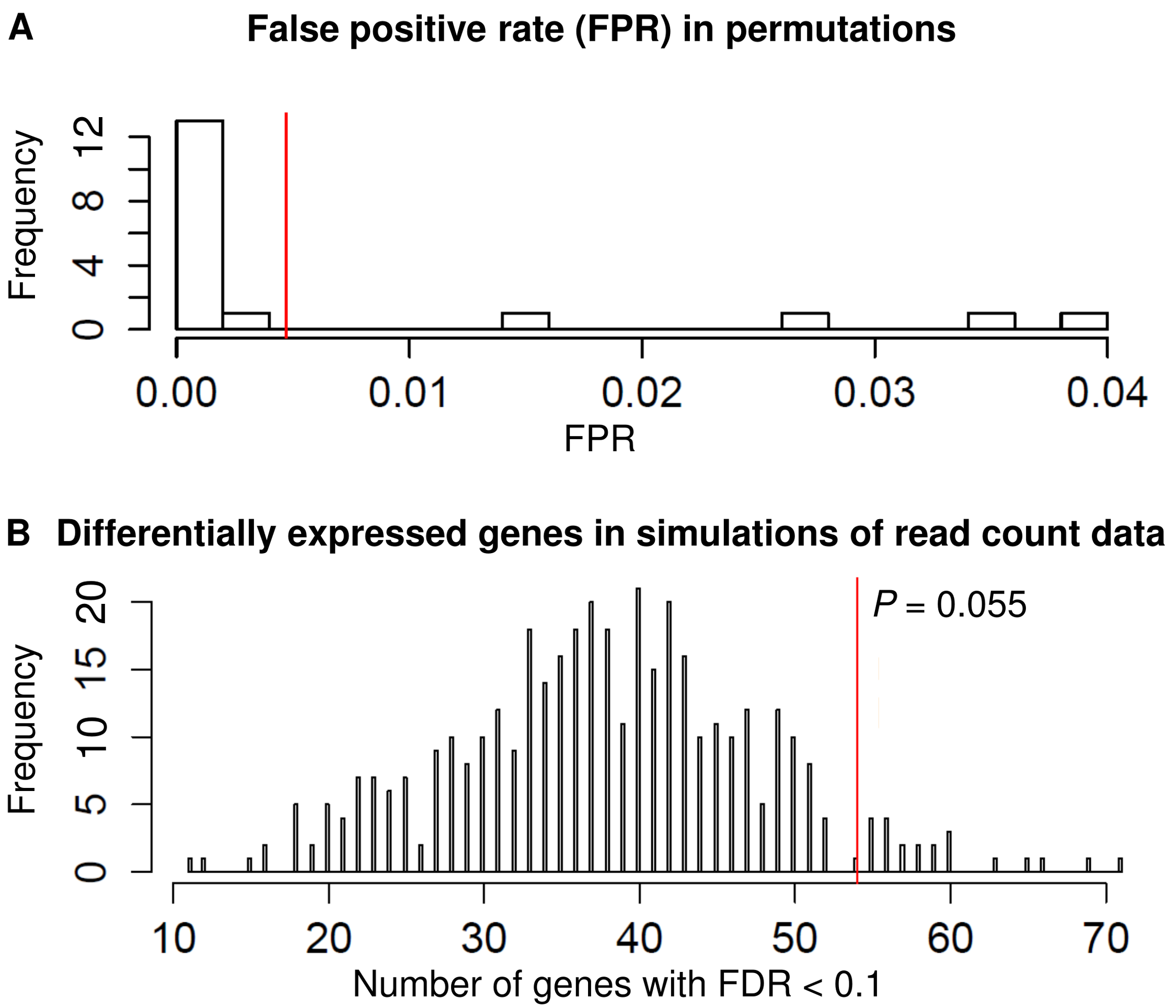

Supplement: S4 Fig — A. Differentially expressed genes detected in 18 permutations of the four Std/Std and Std/Inv samples with RNA-Seq data in which two individuals of each genotype group have been exchanged. False positive rate (FPR) indicates the proportion of genes identified between the resulting groups in each of the permutations, and the red line marks the proportion of differentially expressed genes (0.47%) in the comparison of the four individuals with each inversion genotype. Out of the 18 possible combinations, 14 show a lower number of genes compared to the groups determined by inversion genotype. The remaining four permutations all contain certain pairs of individuals in the two groups compared (NA18621 and NA18973 in one, and NA18632 and NA18951 in the other, where both pairs are formed by individuals with different sex, population, and inversion genotype) and might reflect some difference between these pairs of samples not taken into account. B. Histogram of the number of de-regulated genes in 400 simulations generated with the rnbinom function in R to simulate a negative binomial distribution of the read counts for eight samples with the mean and dispersion of the real data calculated by DESeq2. The red line shows the corresponding number of differentially expressed genes between the Std/Std and Std/Inv analyzed by RNA-Seq, which according to the results of the simulations has a p-value (P) of 0.055. In both the permutations and the simulations the analysis was done with DESeq2 using the same parameters and criteria than in the analysis of real data of S5 Table (FDR < 0.1), with the exception that the sex chromosomes were excluded and only coding genes were taken into account. (TIF) [file pgen.1005495.s004.tif]

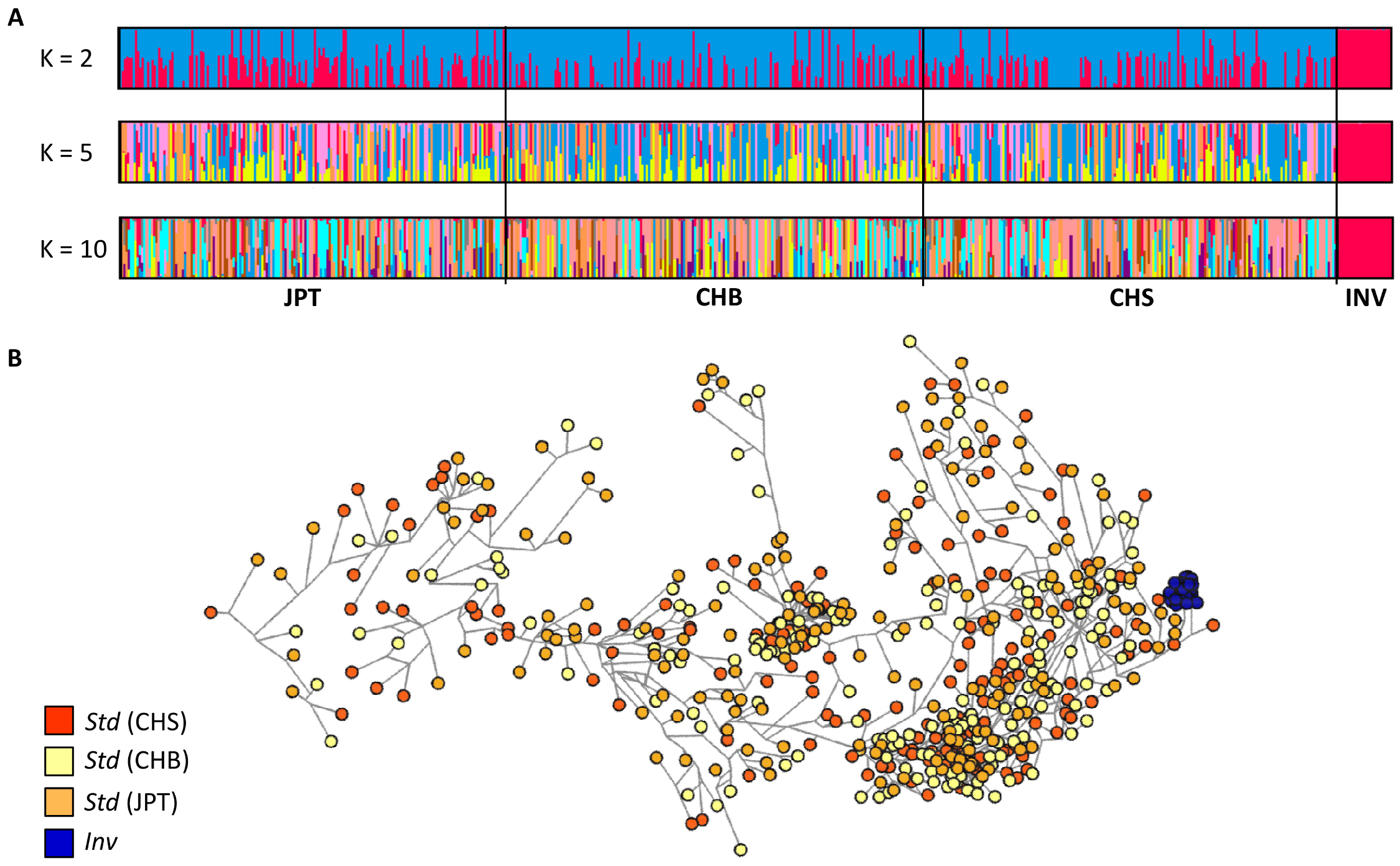

Supplement: S5 Fig — A. Proportion of each chromosome that belongs to different hypothetical ancestral populations. Each chromosome is represented as a vertical line colored according to the proportions of different ancestral components. The number of ancestral components (K) considered in each analysis is indicated at the left. A total of 570 phased chromosomes belonging to three East Asian populations from 1000GP Phase 1 are included. Populations to which Std chromosomes belong are shown below each graph. Inv chromosomes have a single distinctive component and group together although they come from the three analyzed populations. B. Median-Joining network from the same 570 phased chromosomes. Circles correspond to the different haplotypes found for the region of the inversion in the three East Asian populations with Std in different colors and Inv in blue. Circle sizes are proportional to the frequency of each haplotype and the branch length indicates approximately the number of mutations between them [74]. (TIF) [file pgen.1005495.s005.tif]
